# Class A Scavenger Receptor 1 (MSR1) Restricts Hepatitis C Virus Replication by Mediating Toll-like Receptor 3 Recognition of Viral RNAs Produced in Neighboring Cells

**DOI:** 10.1371/journal.ppat.1003345

**Published:** 2013-05-23

**Authors:** Hiromichi Dansako, Daisuke Yamane, Christoph Welsch, David R. McGivern, Fengyu Hu, Nobuyuki Kato, Stanley M. Lemon

**Affiliations:** 1 Division of Infectious Diseases, Department of Medicine, and the Lineberger Comprehensive Cancer Center, The University of North Carolina at Chapel Hill, Chapel Hill, North Carolina, United States of America; 2 Department of Tumor Virology, Okayama University Graduate School of Medicine, Dentistry, and Pharmaceutical Science, Okayama, Japan; McMaster University, Canada

## Abstract

Persistent infections with hepatitis C virus (HCV) may result in life-threatening liver disease, including cirrhosis and cancer, and impose an important burden on human health. Understanding how the virus is capable of achieving persistence in the majority of those infected is thus an important goal. Although HCV has evolved multiple mechanisms to disrupt and block cellular signaling pathways involved in the induction of interferon (IFN) responses, IFN-stimulated gene (ISG) expression is typically prominent in the HCV-infected liver. Here, we show that Toll-like receptor 3 (TLR3) expressed within uninfected hepatocytes is capable of sensing infection in adjacent cells, initiating a local antiviral response that partially restricts HCV replication. We demonstrate that this is dependent upon the expression of class A scavenger receptor type 1 (MSR1). MSR1 binds extracellular dsRNA, mediating its endocytosis and transport toward the endosome where it is engaged by TLR3, thereby triggering IFN responses in both infected and uninfected cells. RNAi-mediated knockdown of MSR1 expression blocks TLR3 sensing of HCV in infected hepatocyte cultures, leading to increased cellular permissiveness to virus infection. Exogenous expression of Myc-MSR1 restores TLR3 signaling in MSR1-depleted cells with subsequent induction of an antiviral state. A series of conserved basic residues within the carboxy-terminus of the collagen superfamily domain of MSR1 are required for binding and transport of dsRNA, and likely facilitate acidification-dependent release of dsRNA at the site of TLR3 expression in the endosome. Our findings reveal MSR1 to be a critical component of a TLR3-mediated pattern recognition receptor response that exerts an antiviral state in both infected and uninfected hepatocytes, thereby limiting the impact of HCV proteins that disrupt IFN signaling in infected cells and restricting the spread of HCV within the liver.

## Author Summary

Persistent hepatitis C virus (HCV) infection is an important cause of fatal cirrhosis and liver cancer in humans. While viral disruption of interferon (IFN) signaling pathways may contribute to the persistence of HCV, IFN-stimulated gene (ISG) expression is often prominent within the infected liver. We show here that this is due, at least in part, to Toll-like receptor 3 sensing of HCV mediated by class A scavenger receptor type 1 (MSR1)-dependent endocytosis and transport of extracellular viral double-stranded RNA (dsRNA) allowing it to be engaged by TLR3 in the late endosome. TLR3 expressed within uninfected cells is capable of sensing HCV infection in neighboring infected cells in a process that is dependent upon the dsRNA-scavenging activity of MSR1, resulting in the induction of a localized functional antiviral response. This contributes to the ISG expression that typifies the chronically-infected liver, as it occurs within cells that do not express HCV proteins that disrupt IFN signaling. TLR3 signaling thus limits the spread of virus within the liver, potentially explaining why only a small fraction of hepatocytes are infected with HCV in vivo.

## Introduction

Hepatitis C virus (HCV) is an hepatotropic, positive-strand RNA virus classified within the *Flaviviridae* family [1]. It is an important human pathogen since most individuals fail to eliminate the virus when first infected. This results in persistent infection and a chronic inflammatory state within the liver that leads over time to clinically significant complications including progressive liver fibrosis, cirrhosis, and hepatocellular carcinoma. The mechanisms underlying these events are only partially understood. The single-stranded RNA (ssRNA) HCV genome encodes a large polyprotein precursor of approximately 3000 amino acid residues. This is cleaved co- and post-translationally into at least 10 mature proteins, at least 3 of which contribute to the virus structure (core, and two envelope proteins, E1 and E2), with the remaining 7 proteins generally considered to be nonstructural in nature (p7, NS2, NS3, NS4A, NS4B, NS5A, and NS5B). NS5B is an RNA-dependent RNA polymerase and the catalytic core of a large macromolecular, membrane-bound replicase complex that directs replication of the viral RNA, producing double-stranded RNA (dsRNA) replication intermediates as well as new viral genomes. These viral RNAs are recognized as pathogen-associated molecular patterns (PAMPs) by innate immune sensors in host cells, but exactly which sequences and how these RNAs are sensed remains only partly elucidated [2].

In general, dsRNAs produced by viruses are recognized by several classes of cellular pattern recognition receptors, including retinoic acid-inducible gene I (RIG-I)-like helicases that are expressed within the cytoplasm, or Toll-like receptors (TLRs), such as TLR-3 that is expressed within and signals from a late endosomal compartment in several different cell types, including hepatocytes [3], [4], [5], [6]. The engagement of these receptors by viral RNAs results in the induction of interferon (IFN)-β synthesis through separate downstream signaling pathways that utilize unique adaptor molecules but result in the common activation of the transcription factors NF-κB and IFN regulatory factor 3 (IRF-3) [reviewed in 7]. This leads in turn to the expression of numerous IFN-inducible genes, including IFN-stimulated gene (ISG)15, ISG56, and others, through both autocrine and paracrine signaling involving the Janus kinase/signal transducer and activator of transcription (JAK/STAT) pathway. RIG-I, and the related cytosolic helicase, myeloid differentiation-associated gene 5 (MDA5) and TLR3 differ substantially in their ligand specificities. RIG-I recognizes short dsRNA molecules and ssRNA with a 5′-triphosphate, while MDA5 recognizes lengthy dsRNA [5], [8]. In addition, the roles played by RIG-I (which is generally constitutively expressed) and MDA5 (IFN-inducible) in recognition of RNA viruses appear to vary by virus type. RIG-I is primarily responsible for innate immune recognition of paramyxoviruses, influenza virus and Japanese encephalitis virus, while MDA5 is thought to be critical for the recognition of encephalomyocarditis virus and possibly other picornaviruses [5]. TLR3 recognizes dsRNA molecules greater than 40–50 bp in length and also mediates the induction of antiviral responses in cells infected with a wide variety of RNA viruses, including West Nile virus, rhinovirus, respiratory syncytial virus, vesicular stomatitis virus, lymphocytic choriomeningitis virus and influenza virus [9], [10]. However, unlike the cytosolic RIG-I-like helicases, TLR3 initiates signaling from within the endosome in a process that is dependent upon acidification of the endosome [11], [12].

In HCV-infected cells, the 5′-triphosphate of genomic RNA is recognized by RIG-I, as is a poly-U/UC segment near the 3′ end of the genome that serves as a PAMP [13], [14]. This results in the induction of an IFN response shortly after infection of cells [14]. However, RIG-I-initiated signaling is subsequently blocked since NS3/4A, the major HCV protease, proteolytically cleaves the essential RIG-I adaptor protein, mitochondrial antiviral signaling protein (MAVS) [14], [15], [16]. The only continuous cell lines that support robust HCV replication are derived from Huh-7 human hepatoma cells, which like many transformed hepatocytes are TLR3 null [17]. However, HCV infection similarly induces an early antiviral response in RIG-I-defective Huh-7.5 cells in which TLR3 expression has been reconstituted [4]. As with RIG-I signaling, TLR3 signaling is also blocked as viral proteins accumulate and NS3/4A mediates cleavage of an essential adaptor protein, in this case Toll-like receptor adaptor molecule 1 (TICAM-1, a.k.a. TRIF) [4], [18]. How TLR3 is able to sense HCV infection is uncertain, since double-stranded HCV RNA is detected primarily adjacent to ER membranes in association with the nonstructural protein, NS5A, or juxtaposed to cytoplasmic lipid droplets [19], while TLR3, as described above, senses dsRNA and initiates signaling only from within late endosomes [11], [12].

One possibility is that dsRNA replication intermediates are released into the extracellular milieu by HCV-infected cells and, like poly(I:C) added to the medium of cultured cells, subsequently sensed by TLR3. Since TLR3 is unable to bind extracellular dsRNA on the cell surface [12], this would require a receptor molecule to direct the endocytosis and trafficking of dsRNA to the late endosome. Scavenger receptors of several different classes, including C-type lectin domain-containing receptors such as the oxidized low density lipoprotein (LDL) receptor (ORL-1), or class A scavenger receptors such as macrophage scavenger receptor 1 (MSR1), have been suggested to fulfill this function, transporting dsRNA from the surface of cells to the endosome where it is sensed by TLR3 [20], [21], [22]. The epidermal growth factor receptor (EGFR) also plays an essential role in TLR3 signaling, but this appears to occur at a later step, following the engagement of TLR3 by dsRNA within the endosome [23].

Here, we show that MSR1 is essential for TLR3 sensing of HCV infection in cells derived from human hepatocytes, and that it mediates the establishment of a localized antiviral response in neighboring, uninfected cells that restricts the replication of virus in cell culture. Since uninfected hepatocytes are not subject to the myriad mechanisms that virally-encoded HCV proteins have evolved to disrupt the induction of IFN responses [2], these observations reveal a mechanism that may explain both the significant levels of ISG expression that typify the HCV-infected liver [24], as well as the fact that only a small minority of hepatocytes are infected by HCV in human liver [25].

## Results

### Replication intermediates trigger TLR3 signaling in HCV-infected cells

**Figure 1:**
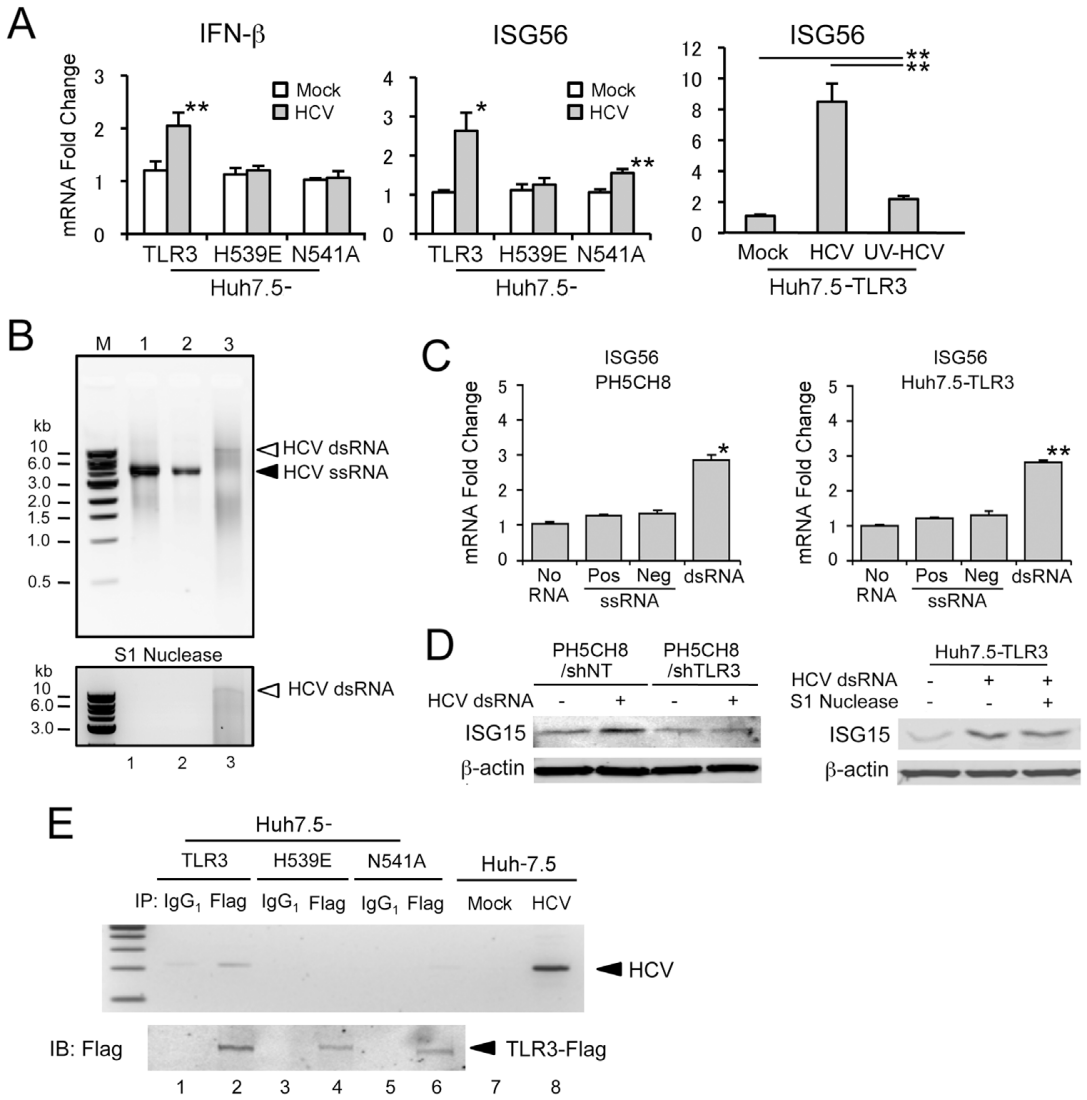
TLR3 senses double-stranded RNA products of HCV replication in human hepatocytes. (**A**) Quantitative RT-PCR analysis of (left) IFN-β and (center and right) ISG56 mRNA in Huh7.5-TLR3 cells infected with HCV. Cells were infected with HCV (HJ3-5) or UV-inactivated HCV at an m.o.i. of 1 for 72 hrs, prior to extraction of RNA and measurement of IFN-β and ISG56 mRNA. Statistical comparisons were made between mock- and HCV-infected cultures (left and center panels), or between samples indicated by bars (right panel): * p≤0.02, ** p≤0.001. (**B**) Agarose gel electrophoresis of in vitro transcribed HCV RNAs (top panel) before or (bottom panel) after S1 nuclease treatment. M: DNA standards ladder; lane 1, positive-strand HCV RNA; lane 2, negative-strand HCV RNA; lane 3, annealed double-stranded HCV RNA. (**C**) Quantitative RT-PCR analysis of ISG56 mRNA in (left) PH5CH8 and (right) Huh7.5/TLR3 cells treated with 50 µg/ml of ssRNA or dsRNA for 6 hrs prior to extraction of RNA. Statistical comparisons were made with cultures not treated with RNA: * p≤0.02, ** p≤0.001. (**D**) (left panel) Immunoblot analysis of ISG15 expression induced by synthetic HCV dsRNA in PH5CH8 cells previously transduced with lentivirus vectors expressing non-targeting (NT) or TLR3-specific shRNAs. β-actin was included as a loading control. (right panel) Similar immunoblots of Huh7.5-TLR3 cells treated with HCV dsRNA, either with or without prior S1 nuclease digestion. In both panels, cell lysates were prepared for immunoblotting 24 hrs after addition of dsRNA. (**E**) HCV RNA co-immunoprecipitates with Flag-TLR3 from lysates of HCV-infected Huh7.5-TLR3 cells. (top panel) Agarose gel electrophoresis showing detection of HCV-specific RNA by RT-PCR in anti-Flag (lanes 2, 4 and 6), or isotype control (mouse IgG_1_, lanes 1, 3 and 5), immunoprecipitates prepared from lysates of Huh7.5-TLR3 cells (lanes 1 and 2), Huh7.5-H539E cells (lanes 3 and 4), or Huh7.5-H539E cells (lanes 5 and 6), infected with HJ3-5 virus (m.o.i. = 1) for 72 hrs. RNA extracted from mock or HCV-infected Huh-7.5 cells was assayed in parallel in the RT-PCR reaction as negative and positive controls, respectively (lanes 7 and 8). (bottom panel) Anti-Flag immunoblots of the respective immunoprecipitates.

While TLR3 is expressed and functional in primary cultures of human hepatocytes, Huh-7 cells and their derivatives have a TLR3-null phenotype [4], [17]. Thus, to establish an HCV-permissive cell line in which we could study viral interactions with the TLR3 signaling pathway, we reconstituted TLR3 expression in Huh-7.5 cells, which are also RIG-I-deficient [4], [26]. Using this, and related cell lines, we demonstrated previously that HCV infection is sensed by TLR3, resulting in an early antiviral response [4]. However, HCV ultimately restricts TLR3 signaling as the NS3/4A protease mediates cleavage of TICAM-1 as viral proteins accumulate in abundance [4], [18]. To further confirm that TLR3 induces a functional antiviral response against HCV, we compared the number of colonies of viable cells originating from TLR3-competent Huh7.5-TLR3 cells transfected with a dicistronic, genome-length HCV replicon that expresses neomycin phosphotransferase [pTat2ANeo/H77S, 27] versus similarly transfected TLR3-null Huh-7.5 cells reconstituted with non-functional TLR3 mutants. As expected, the number of G418-resistant colonies arising from Huh7.5-TLR3 cells was significantly lower than from several related, TLR3-null Huh-7.5 cell lines: Huh-7.5-Vect (empty vector), −ΔTIR (TLR3 lacking the TIR domain required for downstream signaling), and −H539E and −N541A (point mutations in the ectodomain of TLR3 that restrict its ability to bind dsRNA) (Supplementary Fig. S1). Consistent with these results, quantitative real-time PCR demonstrated significant increases in the abundance of IFN-β and ISG56 mRNAs in Huh7.5-TLR3 cells following infection with a laboratory strain of HCV (HJ3-5 virus) (Fig. 1A, left and center panels). Similar increases were not observed in Huh-7.5 cells expressing the signaling-incompetent TLR3 mutants H539E or N541A. Importantly, prior UV inactivation of the virus (UV-HCV) almost completely eliminated the increase in ISG56 mRNA (Fig. 1A, right panel). On the basis of these and our previously published results [4], we conclude that TLR3 senses HCV infection and induces the expression of a functional antiviral state in Huh7.5-TLR3 cells, and that this requires active replication of the virus. This is consistent with a recent report indicating that TLR3-mediated induction of proinflammatory cytokines by HCV also requires active replication of virus [28].

To better understand these results, we examined the ability of in vitro synthesized HCV RNAs to stimulate TLR3 signaling. Since replication of the virus is required to initiate signaling, we reasoned that TLR3 signaling follows its engagement by dsRNA molecules produced by transcription of negative-strand RNA intermediates, rather than multiple structured RNA elements that are known to exist within the 5′ and 3′ untranslated regions as well as in the core and NS5B protein-coding regions of the positive-strand genome [29]. To test this, we synthesized full-length positive- and negative-strand viral RNAs by in vitro transcription using as template an infectious molecular clone of the genotype 2a JFH1 strain (Fig. 1B, upper panel, lanes 1 and 2), and annealed these to produce HCV dsRNA (Fig. 1B, lane 3). As anticipated, the dsRNA products produced by annealing the two ssRNAs were highly heterogenous, resulting in a smear when separated by agarose gel electrophoresis. However, a small amount of what appeared to be full-length dsRNA was generated (Fig. 1B, arrow). While the positive- and negative-sense ssRNAs were degraded by S1 nuclease, this dsRNA was S1 nuclease-resistant, confirming that the complementary in vitro synthesized HCV RNAs anneal to produce dsRNA (Fig. 1B, lower panel). Importantly, only the nuclease-resistant dsRNA induced transcription of ISG56 mRNA when added to the medium of PH5CH8 cells, a T-antigen transformed, human hepatocyte line that naturally expresses TLR3, or Huh7.5-TLR3 cells [30] (Fig. 1C, left and right panels). HCV dsRNA also induced ISG15 protein expression in PH5CH8 and Huh7.5-TLR3 cells (Fig. 1D). Prior transfection of PH5CH8 cells with shRNA targeting TLR3 eliminated the induction of ISG15 protein expression by HCV dsRNA (Fig. 1D, left panel), confirming that ISG15 induction was mediated through TLR3. Prior digestion of the dsRNA with S1 nuclease did not alter the pattern of ISG15 induction (Fig. 1D right panel). Collectively, these data indicate that S1-resistant, double-stranded synthetic HCV RNA is capable of triggering an antiviral response through TLR3, while highly structured RNA elements of the single-stranded genome are not sensed by TLR3. In part, this may be due to the length of the synthetic dsRNAs, which mimic very lengthy dsRNA intermediates produced during replication of the 9.7 kb HCV genome (Fig. 1B) and are much longer than the stable helices present in the ssRNA. While the minimum length of dsRNA required for recognition by the TLR3 ectodomain is on the order of 40 to 50 base pairs [31], we have observed that TLR3 is substantially more responsive to high-molecular weight (1.5–8.0 kb) versus low molecular weight poly(I:C) (0.2–1.0 kb) (Supplementary Fig. S2).

Consistent with the induction of a TLR3-mediated antiviral response by HCV, we detected formation of a TLR3-HCV RNA complex in Huh7.5-TLR3 cells infected with HJ3-5 virus. This was accomplished by immunoprecipitating TLR3 from lysates of infected Huh7.5-TLR3 cells with an anti-Flag antibody, and subjecting RNA extracted from the precipitates to RT-PCR specific for HCV RNA. Viral RNA was readily detected in anti-Flag precipitates prepared from lysates of infected Huh7.5-TLR3 cells (Fig. 1E, lane 2). While somewhat less Flag product was evident in immunoblots of precipitates from cells expressing TLR3-H539E or TLR3-N541A, TLR3 mutants deficient in binding dsRNA [4], HCV RNA was not detected in these precipitates (Fig. 1E, lanes 4 and 6). Taken together, these results indicate that dsRNA produced in membrane-bound replicase complexes during HCV replication is transported to the late endosome where it is engaged by TLR3. Subsequent experiments focused on the mechanism by which dsRNA traffics to the endosome.

### Macrophage scavenger receptor 1 (MSR1) is required for TLR3 engagement by HCV RNA

**Figure 2:**
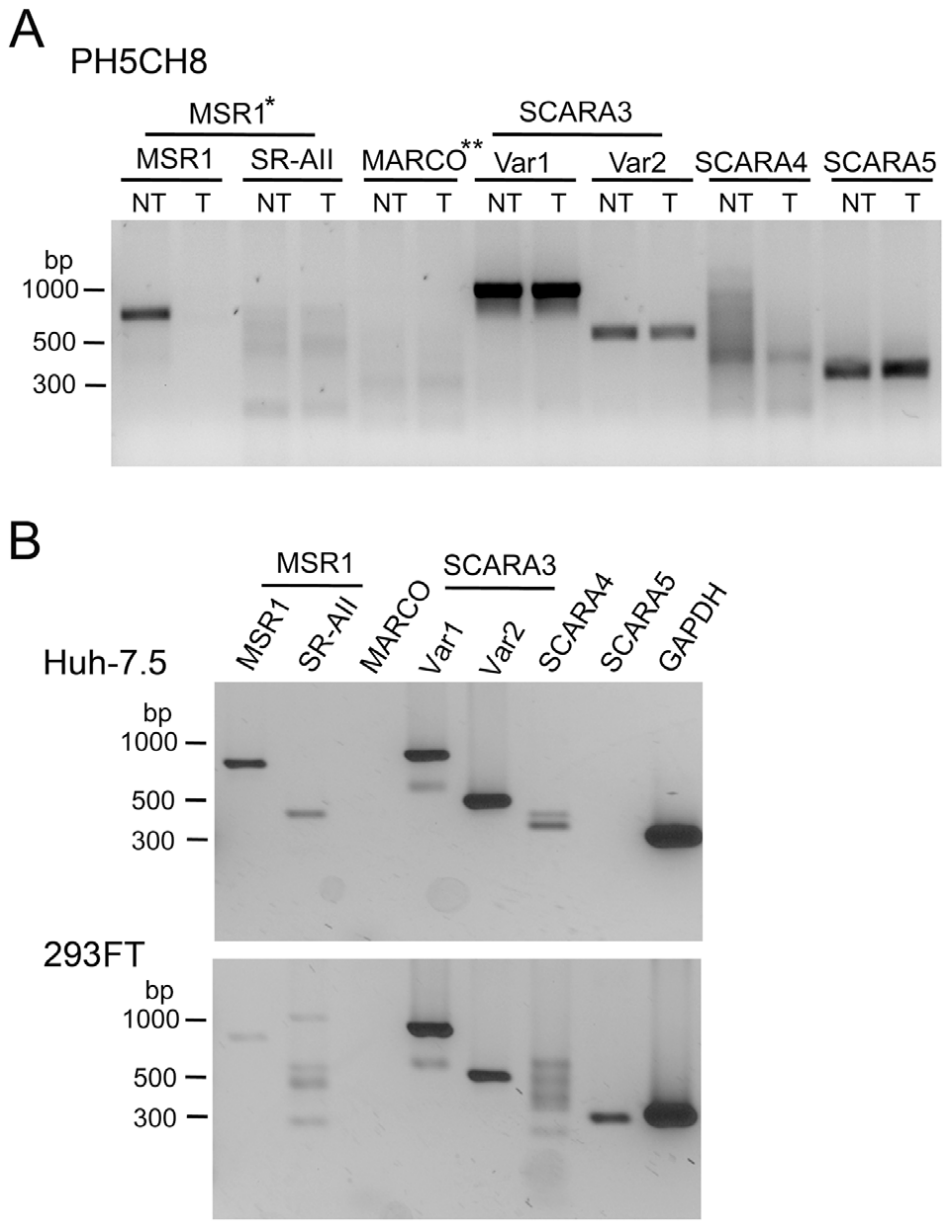
Scavenger receptor class A family transcript profiling of human hepatocyte-derived cell lines. (**A**) RT-PCR detection of class A scavenger receptor mRNAs in total cellular RNAs extracted from PH5CH8 cells transduced to express (“NT”) a non-targeting shRNA (PH5CH8/shNT cells), or (“T”) MSR1-specific shRNA (PH5CH8/shMSR1 cells). The parental PH5CH8 cells are T-antigen transformed normal adult human hepatocytes. Primers specific for MSR1 (SCARA1) transcript variant 1 (MSR1, SR-AI, or CD204) and transcript variant 2 (SR-AII), MARCO (SCARA2), SCARA3 (transcript variants 1 and 2), SCARA4, and SCARA5 (a putative scavenger receptor class A family member) were identical to those used by DeWitte-Orr et al. [22]. PCR was carried through 35 cycles prior to separation of products on an agarose gel. (**B**) Similar class A scavenger receptor transcript profiling of Huh-7.5 cells derived from a human hepatocellular carcinoma, and 293FT cells (Invitrogen). *MSR1 has two transcript variants: variant 1 is commonly referred to as “MSR1”, as we refer to it here, but is otherwise known at SCARA1 or SR-AI. Transcript variant 2 is referred to as SR-AII. See text for additional details. **MARCO, macrophage receptor with collagenous structure, is otherwise known as SCARA2.

Recent studies suggest that class A scavenger receptors, including “macrophage” scavenger receptor 1 (MSR1, class A scavenger receptor type 1, transcript variant 1), are expressed in a variety of cell types and serve as the dominant receptors mediating endocytosis of dsRNA in fibroblasts [20], [22]. Class A scavenger receptors recognize a broad range of ligands including acetylated LDL, and lipopolysaccharide (LPS) produced by Gram-positive bacteria, and have been shown to mediate endocytosis of both ssRNA and dsRNA [22], [32]. We thus hypothesized that dsRNA could be released into the extracellular milieu by hepatocytes infected with HCV, where it could be bound by scavenger receptors expressed on the cell surface and subsequently transported to the endosome for recognition by TLR3. To confirm that MSR1, and possibly other members of the class A scavenger receptor family, are expressed by human hepatocytes, we utilized RT-PCR to ascertain the presence of mRNA transcripts for these proteins in PH5CH8 cells. Transcripts encoding MSR1 (SCARA1, transcript variant 1, referred to generally as “MSR1”), scavenger receptor class A member 3 (SCARA3, transcript variants 1 and 2), SCARA4 (a.k.a. collectin sub-family member 12, COLEC12), and SCARA5 (a putative class A scavenger receptor) transcripts were readily detected (Fig. 2A). Transcripts for MSR1 transcript variant 2 (referred to as “SR-AII”), and MARCO (a.k.a. SCARA2) were not detected, or were present only in very low abundance. MSR1 and SCARA3, variants 1 and 2, expression were also confirmed in Huh-7.5 cells (Fig. 2B, top panel). However, in contrast to PH5CH8 cells, SR-AII transcripts were also evident in Huh-7.5 cells, while SCARA5 transcripts were absent.

**Figure 3:**
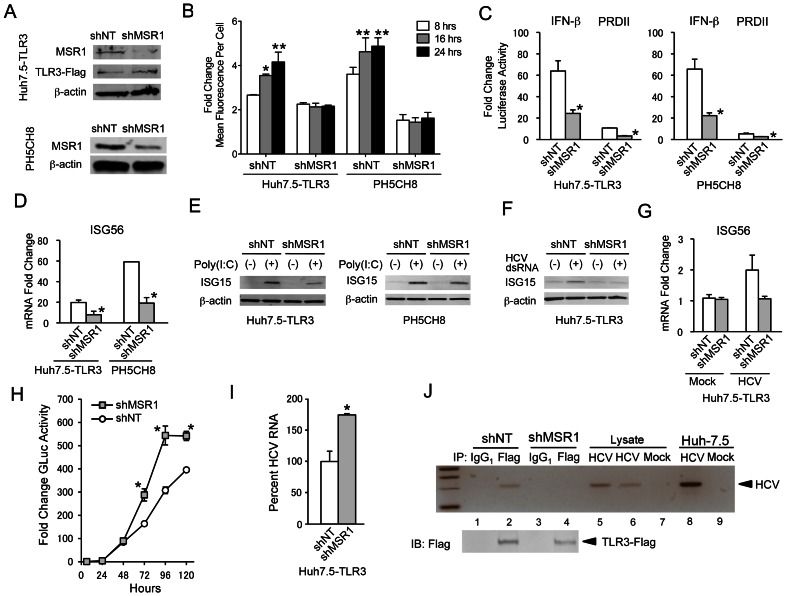
Human macrophage scavenger receptor class A is required for TLR3 signaling triggered by HCV in hepatocytes. (**A**) Immunoblot analysis of MSR1 expression in (top panels) Huh7.5-TLR3 and (bottom panels) PH5CH8 cells transduced with MSR1-specific (shMSR1) or non-targeting control shRNA (shNT) lentiviruses. (**B**) Effect of shRNA-mediated MSR1 depletion on uptake of FITC-labeled poly(I:C) by Huh7.5-TLR3 or PH5CH8 cells. Cells were incubated with 10 µg/ml FITC-labeled poly(I:C) for 8, 16 or 24 hrs before harvest. Cells were washed extensively before fixation and analyzed by flow cytometry. Uptake of FITC-labeled poly(I:C) is expressed as fold-change in mean fluorescence intensity (MFI) compared to mock-treated cells. Statistical comparisons between the MFI at 8 hrs vs. the time indicated were made by two-way ANOVA with Bonferroni correction for multiple comparisons: * p≤0.02, ** p≤0.001. (**C**) Effect of shRNA-mediated MSR1 depletion on TLR3 signaling. IFN-β and PRD-II promoter activation was induced by extracellular poly(I:C) in MSR1-depleted (left) Huh7.5-TLR3 and (right) PH5CH8 cells exposed to 50 µg/ml of high molecular weight poly(I:C) for 6 hrs. Statistical comparisons in this and other panels in this figure were between the shNT and shMSR1-treated cells: * p≤0.02, ** p≤0.001. (**D**) Quantitative RT-PCR analysis of ISG56 mRNA in MSR1-depleted cells treated with poly(I:C) as in panel C. (**E**) Immunoblots of ISG15 expression in MSR1-depleted (left) Huh7.5-TLR3 and (right) PH5CH8 cells treated with poly(I:C) for 24 hrs. (**F**) Immunoblot of ISG15 in MSR1-depleted Huh7.5-TLR3 cells exposed to synthetic HCV dsRNA 50 µg/ml for 24 hrs. (**G**) Quantitative RT-PCR analysis of ISG56 mRNA in Huh7.5-TLR3/shMSR1 or Huh7.5-TLR3/shNT cells infected with HJ3-5 virus at an m.o.i. of 1 for 72 hrs. (**H**) Viral replication assessed by the fold-change in secreted *Gaussia* luciferase in cultures of Huh7.5-TLR3/shMSR1 vs. Huh7.5-TLR3/shNT cells following infection with HJ3-5/GLuc2A virus. The level of *Gaussia* luciferase activity at each time point was calculated relative to that at 6 hrs after infection, which was set at 1. Data are the mean ± s.d. from three independent experiments. The difference in GLuc expression by Huh7.5-TLR3/shMSR1 vs. Huh7.5-TLR3/shNT cells was significant by two-way ANOVA (p = 0.006). Bonferroni post-tests were used to estimate the significance of differences at individual time points: * p≤0.02. (**I**) Quantitative RT-PCR analysis of HCV RNA in Huh7.5-TLR3/shMSR1 vs. Huh7.5-TLR3/shNT cells 72 hrs after infection with HCV. RNA abundance in the Huh7.5-TLR3/shMSR1 was calculated relative to that in Huh7.5-TLR3/shNT, which was set at 100%. (**J**) Co-immunoprecipitation of HCV RNA with Flag-TLR3 in lysates of Huh7.5-TLR3/shMSR1 (lanes 3, 4, and 6) and Huh7.5-TLR3/shNT cells (lanes 1, 2 and 5) infected with HJ3-5 virus 72 hrs previously. Immunoprecipitation was with anti-Flag. See legend to Fig. 1E for additional details.

Consistent with these results, immunoblots demonstrated expression of MSR1 in both Huh7.5-TLR3 and PH5CH8 cells (Fig. 3A). To determine whether it plays a role in TLR3 signaling in these hepatocyte-derived cell lines, we depleted MSR1 by transducing Huh7.5-TLR3 cells (Huh7.5-TLR3/shMSR1 cells) and PH5CH8 cells (PH5CH8/shMSR1 cells) with lentiviruses expressing MSR1-specific shRNA. Immunoblots demonstrated modest depletion of MSR1 in both Huh7.5-TLR3/shMSR1 cells (63% depletion estimated by quantitation of the Odyssey infrared fluorescence signal against β-actin control) and PH5CH8/shMSR1 cells (55% depletion). RT-PCR confirmed MSR1 knockdown in the PH5CH8 cells, and demonstrated that it was specific and without effect on SCARA3, SCARA4, or SCARA5 transcripts (Fig. 2A). Additional studies showed that MSR1 knockdown had no reciprocal effect on the abundance of SR-AII transcripts (data not shown). Although the MSR1 knockdown was relatively inefficient in both cell types, flow cytometry indicated that time-dependent uptake of FITC-labeled high molecular weight (HMW) poly(I:C), a surrogate for HCV dsRNA, was functionally eliminated in MSR1-depleted cells compared to control cells (Fig. 3B). The high degree of inhibition of poly(I:C) uptake was surprising, given the rather modest degree of MSR1 depletion evident in either cell type (Fig. 3A). It suggests that a threshold level of expression is required for efficient dsRNA uptake, possibly because class A scavenger receptors function as multimeric complexes. IFN-β and NF-κB-responsive PRDII promoter activities were also decreased in MSR1-depleted cells compared to control cells when stimulated by the addition of poly(I:C) to the medium (Fig. 3C), as was poly(I:C) induction of ISG56 (Fig. 3D) and ISG15 (Fig. 3E) expression. These results indicate that MSR1 is required for poly(I:C) uptake and optimal induction of TLR3-mediated signaling by poly(I:C) in human hepatocytes.

Next, we examined whether MSR1 is required for the recognition of HCV-specific dsRNA through TLR3. As with poly(I:C), MSR1 depletion eliminated the induction of ISG15 or ISG56 by synthetic HCV dsRNA added to the medium bathing Huh7.5-TLR3 cells (Fig. 3F and G). We also demonstrated that MSR1 functions in the induction of TLR3-mediated antiviral responses to HCV infection by monitoring viral replication in Huh7.5-TLR3/shNT cells and Huh7.5-TLR3/shMSR1 cells following infection with HJ3-5/GLuc2A virus [33] that expresses the secreted reporter protein, *Gaussia princeps* luciferase (GLuc). Replication was assessed by measuring secreted GLuc activity and by qRT-PCR analysis of intracellular HCV RNA abundance. Both GLuc activity (Fig. 3H) and HCV RNA abundance (Fig. 3I) were significantly increased in MSR1-depleted TLR3-expressing cells compared to control cells transduced with shNT, suggesting at least partial release from a TLR3-mediated antiviral response in these cells. Consistent with a role for MSR1 in transporting HCV RNA to the endosome where it can engage TLR3, HCV RNA was no longer detected in immunoprecipitates of TLR3 prepared from lysates of infected, MSR1-depleted cells (Fig. 3J, lane 4 vs. lane 2).

**Figure 4:**
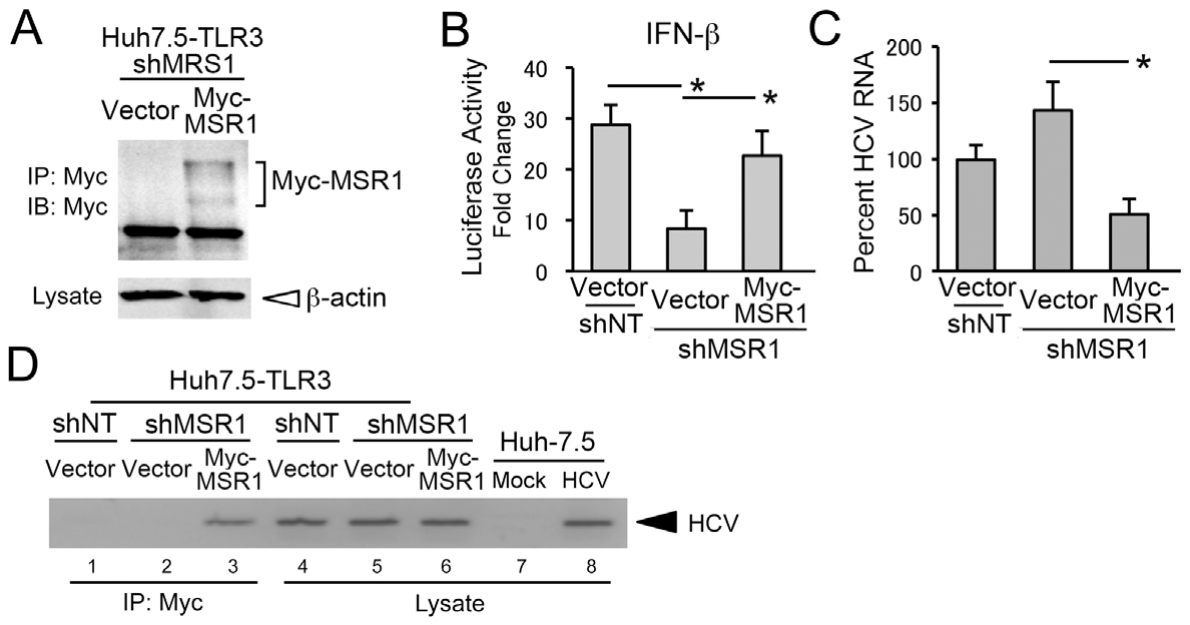
Reconstitution of MSR1 expression in MSR1-depleted cells restores TLR3 signaling triggered by HCV. (**A**) Reconstitution of MSR1 expression in Huh7.5-TLR3/shMSR1 cells. Myc-tagged MSR1 was stably expressed by retroviral transfer of a shMSR1-resistant vector (pCX4bsr/Myc-MSR1). Lysates were immunoprecipitated with anti-Myc, then subjected to immunoblotting as described in Materials and Methods. “Vector” = cells transduced with empty vector. (**B**) Restoration of poly(I:C) induction of IFN-β promoter activity by ectopically expressed Myc-MSR1 in Huh7.5-TLR3/shMSR1 cells. Cells were co-transfected with pCX4bsr/Myc-MSR1 (vs. empty vector), pIFN-β-Luc and pRL-CMV (internal control reporter) and cultured for 24 h, then treated with poly(I:C) (50 µg/ml) for 6 hrs prior to lysis and luciferase assay. Huh7.5-TLR3/shNT cells, transfected with empty vector, were included as a positive control. (**C**) Quantitative RT-PCR analysis of HCV RNA in Huh7.5-TLR3/shMSR1 cells stably expressing Myc-MSR1 (vs. empty vector) 72 hrs after infection with HJ3-5 virus at an m.o.i. of 1. (**D**) Co-immunoprecipitation of HCV RNA with Myc-MSR1 in lysates of Huh7.5-TLR3/shMSR1 cells stably expressing Myc-MSR1 (lanes 3 and 6) vs. empty vector (lanes 2 and 5). Cells were infected with HJ3-5 virus (m.o.i. = 1) 72 hrs prior to lysis. Immunoprecipitation was with anti-Myc, demonstrating association of HCV RNA with Myc-MSR1. See legend to Fig. 1E for further details.

To exclude the unlikely possibility that these results might in some way reflect an off-target effect of shMSR1, we reconstituted MSR1 expression in Huh7.5-TLR3/shMSR1 cells by ectopic expression of Myc-tagged MSR1 using an expression vector lacking the 5′ untranslated RNA (UTR) segment of the endogenous MSR1 mRNA targeted by shMSR1 (Fig. 4A). As we anticipated, poly(I:C) stimulation of IFN-β promoter activity was substantially restored by stable expression of Myc-MSR1 in Huh7.5-TLR3/shMSR1 cells (“Myc-MSR1 cells”) (Fig. 4B). In addition, HCV RNA replication levels were reduced in HCV-infected Myc-MSR1 cells compared to Huh7.5-TLR3/shMSR1 cells (Fig. 4C). Co-immunoprecipitation experiments using an anti-Myc antibody also demonstrated an association between Myc-MSR1 and HCV RNA in HCV-infected Myc-MSR1 cells (Fig. 4D, lane 3).

Collectively, the results shown in Figs. 3 and 4 demonstrate that MSR1 plays an essential role in the transport of double-stranded HCV RNA to TLR3, which engages this ligand in endosomes [4], [11], [12] to initiate signaling triggered by HCV infection.

### A positively-charged region within the collagen superfamily domain of MSR1 is required for TLR3 signaling

**Figure 5:**
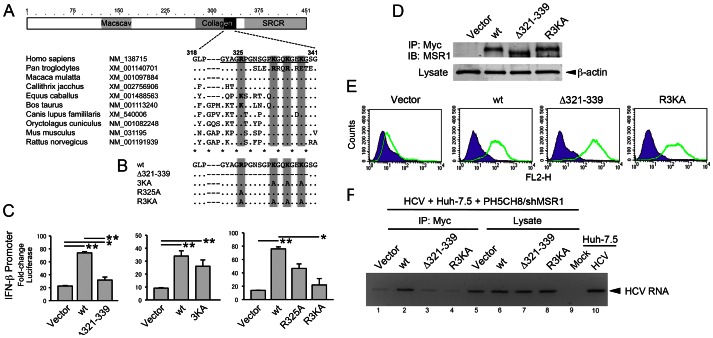
Poly(I:C) induced TLR3 signaling is dependent upon a conserved collagen superfamily domain in MSR1. (**A**) (top) Domain architecture of the 451 a.a. human MSR1 protein showing the location of the collagen superfamily domain that contains multiple G-X-Y repeats that form a triple helix [58]. (bottom) Alignment of human MSR1 with other mammalian MSR1 proteins near the C-terminus of the collagen domain, showing multiple conserved basic residues. GenBank accession numbers are shown for each. “Macscav” = conserved macrophage scavenger receptor domain; “SRCR” = Cys-rich scavenger receptor superfamily domain. (**B**) Myc-MSR1 mutants constructed within the pCX4bsr/Myc-MSR1 plasmid. (**C**) Poly(I:C) stimulated IFN-β promoter activity in MSR1-depleted Huh7.5 TLR3/shMSR1 cells transiently expressing wt or mutant Myc-MSR1: (left) Myc-MSR1/Δ321–339 (deletion of underlined sequence in panel A), (center) Myc-MSR1/3KA, or (right) Myc-MSR1/R325A and Muc-MSR1/R3KA. Cells were co-transfected with Myc-MSR1 expression vectors, pIFN-β-Luc and pRL-CMV (internal control), cultured for 24 h, then treated with poly(I:C) (50 µg/ml) for 6 hrs before to lysis and luciferase assay. (**D**) Immunoblots showing expression of wt Myc-MSR1 and related mutants in PH5CH8/shMSR1 cells. Lysates were precipitated with anti-Myc antibody prior to anti-Myc immunoblot. (**E**) Flow cytometry histograms showing expression of Myc-MSR1 (wt) and indicated mutants on the surface of PH5CH8 cells (green lines). Purple curves indicate cell-surface staining with isotype immunoglobulins. “FL2-H” = fluorescent intensity. (**F**) Co-immunoprecipitation analysis of the association of HCV RNA with wt versus mutant Myc-MSR1 proteins in PH5CH8/shMSR1 cells co-cultured with Huh7.5 cells infected with HJ3-5 virus (m.o.i. = 1) for 72 hrs. RNA was extracted anti-Myc precipitates from lysates of co-cultures of infected Huh7.5 cells and PH5CH8/shMSR1 cells stably transduced with empty vector (lane 1 and 5), wt Myc-MSR1 (lane 2 and 6), Δ321–339 (lane 3 and 7), or R3KA (lane 4 and 8). See legend to Fig. 1E for further details.

The domain architecture of MSR1 (Fig. 5A) includes a conserved collagen superfamily domain with approximately 20 Gly-X-Y repeats that are predicted to form a collagen-like, triple-helical structure [34] (Fig. 5A). This domain is required for the binding of acetylated LDL to the bovine homolog of MSR1 [29], but its role in MSR1 recognition of dsRNA is unknown. We postulated that a series of positively-charged residues located within the carboxy terminus of the collagen superfamily domain, between amino acids (a.a.) 325–338 of human MSR1, could provide for interactions with the negatively-charged sugar-phosphate backbone of dsRNA, and thus be important for MSR1-mediated transport of dsRNA to TLR3. To test this hypothesis, we constructed a series of Myc-MSR1 expression vectors with deletion of a.a. 321–339 (Myc-MSR1/Δ321–339), or alanine substitutions at one or more of the following positively-charged residues: Arg^325^, Lys^332^, Lys^335^ and Lys^338^ (Fig. 5B). With the exception of Lys^338^, that is Glu in the chimpanzee (*Pan troglodytes*), a positively-charged side chain is conserved at each of these positions in MSR1 homologs from a wide variety of mammalian species (Fig. 5A). Consistent with an essential role for the carboxy terminus of the collagen superfamily domain in dsRNA trafficking, transient expression of Myc-MSR1/Δ321–339 failed to rescue poly(I:C) induction of IFN-β promoter activity in the MSR1-depleted Huh7.5-TLR3/shMSR1 cells (Fig. 5C, left panel). While Ala substitutions at Lys^332^, Lys^335^ and Lys^338^ (Myc-MSR1/3KA) or at Arg^325^ (Myc-MSr1/R325A) caused only a modest reduction in the ability of the wt Myc-MSR1 to rescue signaling (Fig. 5C, center and right panels), Ala substitutions at all 4 positions (Myc-MSR1/R3KA) resulted in a nearly complete loss of the ability to rescue signaling in Huh7.5-TLR3/shMSR1 cells exposed to poly(I:C) (Fig. 5C, right panel). The mutants were expressed at high levels (Fig. 5D), and flow cytometry indicated that the Myc-MSR1/R3KA and Myc-MSR1/Δ321-229 mutants traffic to the cell surface (Fig. 5E). Thus, the loss of signaling cannot be explained by the MSR1 mutants being improperly processed, aberrantly degraded, or not transported to the cell surface. The results are consistent with a loss of dsRNA-binding capacity by Myc-MSR1/Δ321–339 and Myc-MSR1/R3KA, and suggest that the positively-charged residues in the MSR1 collagen superfamily domain are involved in dsRNA transport and required for TLR3 signaling.

To confirm that these mutations do in fact ablate the ability of MSR1 to bind HCV RNA replication intermediates, we assessed the association of wt Myc-MSR1, Myc-MSR1/Δ321–339 and Myc-MSR1/R3KA with HCV RNA in co-immunoprecipitation experiments (Fig. 5F). HJ3-5 virus-infected Huh-7.5 cells were co-cultured with MSR1-depleted PH5CH8/shMSR1 cells in which stable expression of each of these Myc-MSR1 mutants (or empty vector) had been established. Cell lysates were immunoprecipitated with anti-Myc antibody, and RNA extracted from the immunoprecipitates assayed by HCV-specific RT-PCR. The anti-Myc immunoprecipitate prepared from cells expressing wt Myc-MSR1 was significantly enriched in HCV RNA (Fig. 5F, lane 2) compared to immunoprecipitates prepared from cells expressing either the Δ321–339 or R3KA mutants (lanes 3 and 4), or empty vector (lane 1). Collectively, these results reveal that conserved positively-charged residues within the carboxy terminus of the collagen superfamily domain of MSR1 are essential for TLR3-mediated responses to dsRNA, and that they contribute to a dsRNA-binding domain in MSR1.

### MSR1 facilitates TLR3 recognition of HCV infection in neighboring cells

Since infected Huh-7.5 cells served as the source of viral RNA bound by Myc-MSR1 expressed within the PH5CH8/shMSR1 cells in the co-culture experiment shown in Fig. 5, these results suggest that TLR3, through the dsRNA-scavenging functions of MSR1, is capable of sensing the presence of HCV infection in adjacent cells. This is an important observation, as previous studies demonstrating TLR3 sensing of HCV infection by hepatocytes [4] have not distinguished between the sensing of replication intermediates produced within the same cell versus those released into the extracellular milieu from neighboring infected cells.

**Figure 6:**
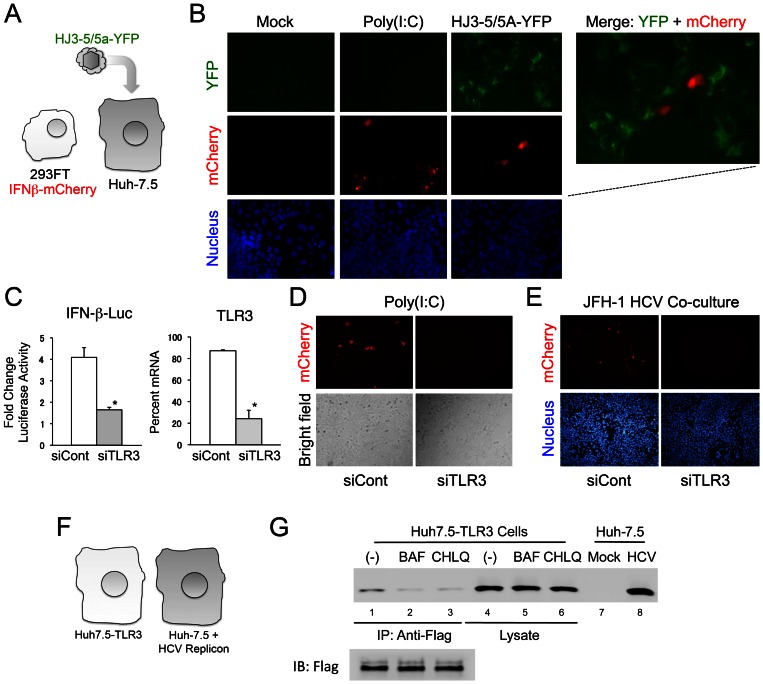
TLR3 expressed in 293-hTLR3 cells senses HCV replication in adjacent human hepatocytes. (**A**) Experimental design, showing co-culture of HCV-nonpermissive, TLR3-competent 293FT/IFN-β-mCherry cells expressing mCherry under control of the IFN-β promoter with Huh-7.5 cells that are HCV permissive and TLR3 incompetent. Cells were infected with HJ3-5/5A-YFP virus that expresses YFP as a fusion with NS5A. (**B**) Immunofluorescence microscopy demonstrating induction of mCherry expression in 293FT/IFN-β-mCherry + Huh-7.5 cell co-cultures upon stimulation with poly(I:C) or infection with HJ3-5/NS5A-YFP virus. HCV replication was visualized by YFP expression and present in cells adjacent to those expressing mCherry in the two-color merged images at the far right. Nuclei were visualized by DAPI counterstain. (**C**) siRNA-mediated depletion of TLR3 significantly reduces poly-(I:C)-induced activation of the IFN-β promoter in 293FT/IFN-β-mCherry cells. (*left*) Fold-change in luciferase activity induced by extracellular poly-(I:C) in 293FT/IFN-β-mCherry cells transfected with TLR3-specific (siTLR3) or control (siCont) siRNAs. The cells were transduced with an IFN-β-Luc reporter plasmid. * p≤0.02. (*right*) qRT-PCR detection of TLR3-specific mRNA in cells transfected with the siTLR3 or siCont siRNAs. * p≤0.02. (**D**) Poly-(I:C)-induced expression of mCherry in 293FT/IFN-β-mCherry cells is ablated by prior transfection with TLR3-specific siRNA. (**E**) siRNA-mediated depletion of TLR3 eliminates mCherry expression by 293FT/IFN-β-mCherry cells placed in co-culture with HCV-infected Huh-7.5 cells. 293FT/IFN-β-mCherry cells were transfected with siTLR3 or siCont siRNAs 3 days prior to being placed in co-culture with Huh-7.5 cells infected with genotype 2a JFH-1 virus. (**F**) Experimental design, showing Huh7.5-TLR3 cells co-cultured with Huh7.5 cells supporting replication of a genome-length genotype 1a HCV RNA replicon. (**G**) Co-immunoprecipitation analysis of the association of HCV RNA with Flag-TLR3 in co-cultures of Huh7.5-TLR3 and HCV replicon cells (panel F) after treatment with bafilomycin (1.0 nM, lanes 2 and 5) or chloroquine (5.0 µM, lanes 3 and 6) for 72 hrs. RNA was extracted from anti-Flag precipitates prepared from lysates of the co-cultured cells and subjected to HCV-specific RT-PCR. See legend to Fig. 1E for further details.

To further explore this phenomenon, we generated cell lines that could serve as either infected “inducer” cells or TLR3-competent “sensor” cells in co-culture experiments (Fig. 6A). The inducer cells were Huh-7.5 cells infected with HJ3-5/5A-YFP virus, which expresses a fusion of the NS5A protein with YFP [35], thereby allowing infected cells to be identified by fluorescence microscopy. These inducer cells are not competent for either RIG-I or TLR3 signaling [17], [26]. The sensor cells were 293FT/IFN-β-mCherry cells, human embryonic kidney cells expressing endogenous TLR3 and the fluorescent reporter protein, mCherry, under the control of the IFN-β promoter. These 293FT/IFN-β-mCherry cells express a low, but readily detectable level of MSR1 transcripts (Fig. 2B, lower panel). Importantly, they are nonpermissive for HCV replication. As expected, co-culture of mock-infected Huh-7.5 cells and the 293FT/IFN-β-mCherry cells resulted in no detectable YFP or mCherry signal (Fig. 6B, left set of panels). However, mCherry expression could be induced by the addition of poly(I:C) to the medium bathing these co-cultured cells (Fig. 6B, second set of panels from the left). Infection of the co-cultured cells with HJ3-5/5A-YFP virus also resulted in the induction of mCherry in the 293FT/IFN-β-mCherry cells (Fig. 6B, right panels and merged image). The cells expressing mCherry lacked detectable YFP fluorescence, confirming that they were not infected. Similar results were obtained with 293/hTLR3-IFN-β-mCherry cells, which are engineered to overexpress TLR3, and also express mCherry under control of the IFN-β promoter (Supplementary Fig. S3). Prior transfection of 293FT/IFN-β-mCherry cells with siRNA targeting TLR3 significantly reduced poly-(I:C)-induced IFN-β promoter activity (Fig. 6C) and mCherry expression (Fig. 6D), confirming that dsRNA sensing by TLR3 activates the IFN-β promoter (and mCherry expression) in these cells. Similarly, siRNA-mediated depletion of TLR3 in 293FT/IFN-β-mCherry cells largely eliminated mCherry expression when the cells were co-cultured with HCV-infected Huh-7.5 cells (Fig. 6E). These results indicate that TLR3 is capable of sensing HCV infection in neighboring cells, presumably via MSR1-mediated transport of extracellular dsRNA to the endosome where TLR3 is expressed.

In separate experiments, HCV RNA co-immunoprecipitated with Flag-TLR3 when Huh7-TLR3 cells were co-cultured with Huh-7.5 cells containing an HCV replicon RNA (Fig. 6F and G), providing additional evidence that HCV RNA produced in adjacent cells may serve as ligand for TLR3. Consistent with the fact that TLR3 signaling is initiated only in the endosome, not at the cell surface [12], and more specifically that acidification of the endosome is required for TLR3 signaling in Huh7.5-TLR3 cells [4], treatment of these co-cultured cells with bafilomycin (1 nM) or chloroquine (5 µM) substantially reduced the amount of HCV RNA that co-immunoprecipitated with TLR3 (Fig. 6G).

**Figure 7:**
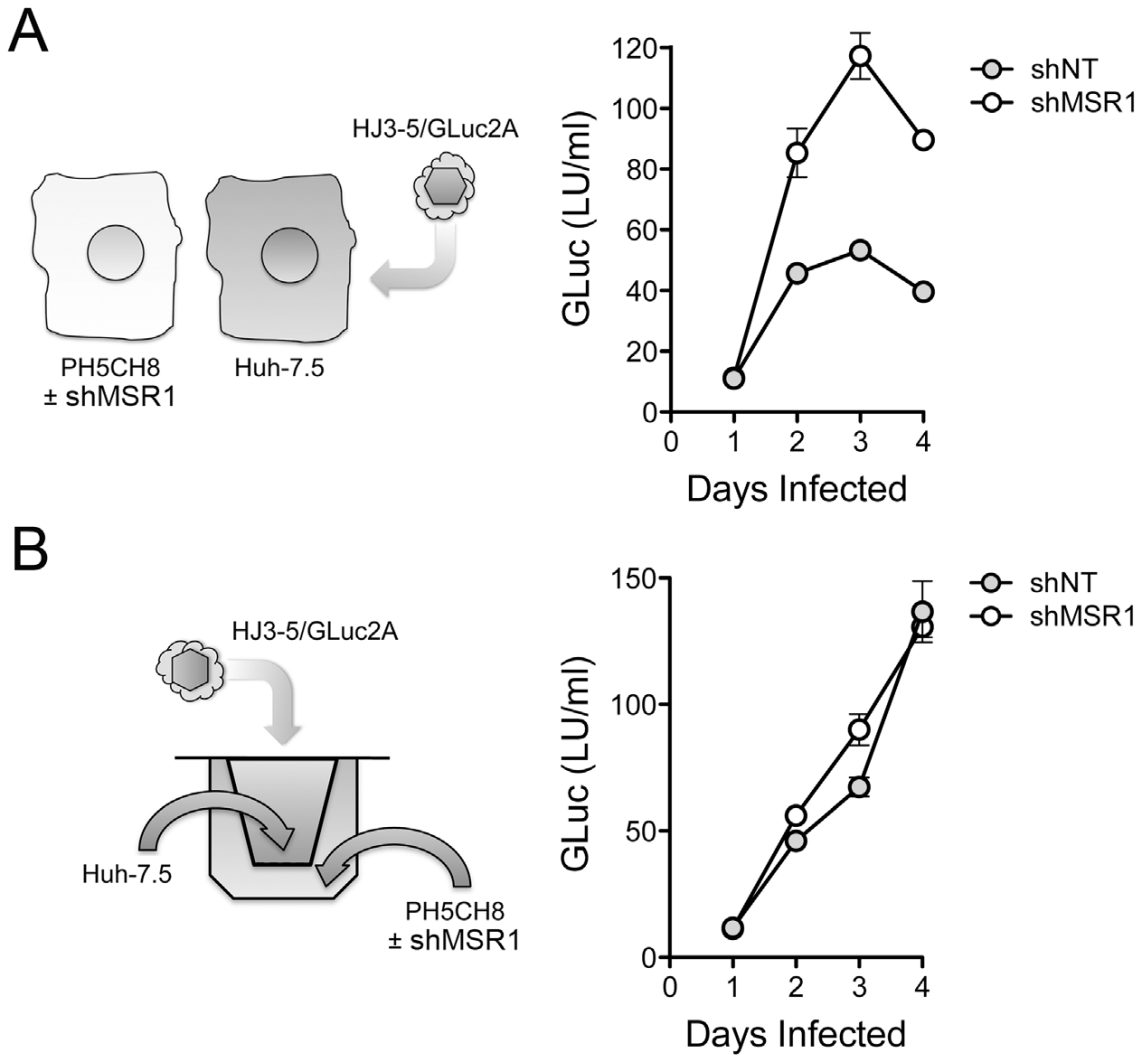
MSR1-dependent TLR3 sensing of HCV infection in neighboring cells restricts viral replication. (**A**) (*left*) Design of co-culture experiments in which HCV-nonpermissive, but TLR3-competent, PH5CH8 cells were co-cultured with HCV-permissive, but RIG-I and TLR3-incompetent, Huh-7.5 cells infected with HJ3-5/GLuc2A, a reporter virus that expresses GLuc as a component of its polyprotein. Huh-7.5 cells were infected with virus for 6 hrs prior to being split and added to either MSR1-depleted PH5CH8/shMSR1 or control PH5CH8/shNT cells (Fig. 3A) at a ratio of 1∶2 to establish co-cultures. (*right*) *Gaussia* luciferase activity in supernatant fluids of HJ3-5/GLuc2A-infected co-cultures. Data shown are means ± s.d. from 3 replicate cultures and are representative of repeat, independent experiments. The difference in GLuc expression from shMSR1- vs. shNT-treated cells was significant by two-way ANOVA (p<0.0001). (**B**) (*left*) Co-cultures of PH5CH8/shMSRI (or control PH5CH8/shNT) cells and HJ3-5/GLuc2A virus-infected Huh-7.5 cells in which the cell types are separated by a semi-permeable membrane (Transwell system). (*right*) *Gaussia* luciferase activity in supernatant fluids of HJ3-5/GLuc2A virus-infected Huh-7.5 cells separated from the PH5CH8 cells by a semi-permeable membrane. Data shown are means ± s.d. from 3 replicate cultures. Unlike Huh-7.5 cells cultured in close continuity with PH5CH8 cells (panel A), there is no restriction to HCV replication when the cell types are separated by a permeable membrane. The difference in GLuc expression from shMSR1- vs. shNT-treated cells was not significant by two-way ANOVA (p = 0.20).

To demonstrate that TLR3 signaling induced within cells adjacent to those infected results in functional antiviral activity, as well as to formally demonstrate a role for MSR1 in this process, we replaced the 293 sensor cells with PH5CH8 cells (Fig. 7A, left). These T antigen-transformed, TLR3-competent human hepatocytes [17] are nonpermissive for HCV infection due in part to a lack of expression of miR-122 (D. Yamane and S.M. Lemon, unpublished data), an essential host factor for HCV replication [36]. We infected Huh-7.5 cells with HJ3-5/GLuc2A virus, and 6 hrs later split the culture to create co-cultures of HCV-infected Huh-7.5 cells and either MSR1-depleted PH5CH8 cells or PH5CH8 cells transduced with the non-targeting control, shNT (Fig. 2A). HCV replication in the co-cultures was monitored over time by measuring GLuc activity in the culture supernatant fluids (Fig. 7A, right). Our expectation was that TLR3-dependent signaling should be triggered in PH5CH8 cells when placed in co-culture with infected Huh-7.5 cells (that lack both TLR3 and RIG-I-mediated signaling) [17], [26], and that this would result in an antiviral response capable of restricting HCV replication in the Huh-7.5 cells through paracrine signaling. This was precisely what we observed. Secreted GLuc activity, which is proportionate to the replication of the reporter virus, was consistently reduced by 50% or more when the infected cells were co-cultured with PH5CH8/shNT versus PH5CH8/shMSR1 cells (Fig. 7A, right). Since some laboratory strains of HCV induce apoptosis in Huh-7.5 cells [37], we determined the proportion of Huh-7.5 cells undergoing apoptosis after 4 days of infection with HJ3-5/GLuc2A virus. These studies demonstrated no increase in the proportion of Huh-7.5 cells expressing detectable cleaved caspase 3 protein or demonstrating TUNEL fluorescence (Supplementary Fig. S4). Thus, apoptosis of infected cells is not required for sensing of infection by adjacent, TLR3 competent hepatocytes.

To gain further insight into the MSR1-mediated restriction of HCV replication in neighboring cells, we assessed the replication of HJ3-5/GLuc2A virus in Huh-7.5 cells that were separated from PH5CH8/shMSR1 (or PH5CH8/shNT) cells by a permeable membrane in Transwell culture dishes (Fig. 7B, left). Under these conditions, MSR1 depletion in the TLR3-competent PH5CH8 cells had no impact on the rate of HCV replication in the Huh-7.5 cells (Fig. 7B, right). These results indicate a need for close positioning and possibly direct cell-cell contact for MSR1-dependent sensing of infection in neighboring hepatocytes. Despite the restriction imposed on virus replication in Huh-7.5 cells by PH5CH8 cells, we were unable to detect IFN-β in media from the direct co-cultures (Fig. 7A) using an ELISA with a level of detection of approximately 10 pg/ml. Collectively, these data show that the antiviral response induced by MSR1-dependent recognition of viral RNA produced in neighboring cells is functional and restricts replication in the infected cell, but that this effect is limited in magnitude and highly localized.

## Discussion

The class A scavenger receptors comprise a diverse family of 5 homotrimeric, single-pass type II membrane proteins that bind to and facilitate the cellular import of a broad range of ligands, including acetylated LDL, bacterial cell wall constituents, and both ssRNA and dsRNA [20], [22], [32]. While their expression has been considered previously to be restricted to cells of myeloid origin, primarily macrophages (hence the name, “macrophage scavenger receptor”), more recent data suggest that members of this receptor family are expressed more ubiquitously and are present on the surface of a variety of cell types [22]. The various members of the family differ in length, domain architecture, ligand specificity and function, but have been recognized increasingly to play important roles in innate immune signaling. Several class A scavenger receptors, in particular MARCO (or SCARA2), and SCARA4 (collectin-12), function in innate immune recognition of bacterial infections, while MSR1 (SCARA1, SR-AI, or CD204) has been shown recently to contribute to antiviral responses evoked by extracellular dsRNA [reviewed in 22]. Mice with genetic deficiency of the homolog of MSR1 demonstrate increased susceptibility to infection with herpes simplex virus [38], while MSR1 is required for induction of TLR3-mediated signaling in monocytes exposed to human cytomegalovirus [39].

DeWitte-Orr et al. [22] have recently suggested that the family of class A scavenger receptors represent the major receptors for dsRNA on the surface of fibroblasts, and that they act in a cooperative fashion to deliver dsRNA to both endosomal TLR3 as well as RIG-I-like helicases expressed within the cytoplasm of these cells. The data we present here provide additional support for this general conclusion, but show with greater specificity that MSR1 is the dominant surface receptor for dsRNA in human hepatocyte-derived cell lines. While DeWitte-Orr et al. [22] found that selective knockdown of any one member of the class A scavenger receptor family (including MSR1) had no effect on dsRNA uptake or poly(I:C)-stimulated ISG expression, we found that a relatively low efficiency knockdown of MSR1 only profoundly disrupts the ability of extracellular poly(I:C) to stimulate IFN-β promoter activity and ISG expression in both Huh7.5-TLR3 cells and PH5CH8 cells (Fig. 3). In all of our studies, these two cell lines behaved similarly. This is important, as Huh-7 cells, from which Huh7.5-TLR3 cells are derived, originate from an hepatocellular carcinoma. In contrast, the PH5CH8 cell line was established by transformation of non-neoplastic human hepatocytes with the large T antigen of simian virus 40 [30]. These cells, like primary hepatocytes, express TLR3 endogenously and are stimulated to produce an IFN response when exposed to extracellular poly(I:C) [4]. Although hepatocytes express several members of the class A scavenger receptor family (Fig. 2), the fact that depletion of MSR1 alone disrupts this response suggests that MSR1 is uniquely required for the uptake and transport of extracellular dsRNA so that it may be sensed by TLR3 in these cells.

In addition to showing that MSR1 expression is required for TLR3-mediated responses to poly(I:C) or infectious challenge with HCV in hepatocyte-derived cells, our data show that MSR1 is physically associated with viral RNA, even when it is produced by ongoing RNA replication in neighboring cells (Fig. 6F and G), and that MSR1 expression is required for TLR3 to bind HCV RNA as ligand (Fig. 3J). We show directly that MSR1 forms a complex with HCV RNA (Fig. 4D), and identify several conserved basic residues within the carboxyl terminus of the collagen superfamily domain that are required for dsRNA uptake by MSR1 and TLR3-mediated signaling in hepatocytes (Fig. 5). There are three alternatively spliced isoforms of MSR1 in humans, only two of which (type I and type II) are expressed on the plasma membrane and facilitate endocytosis of ligands [40]. While the type I isoform is 451 a.a. in length, type II MSR1 is only 358 a.a. However, the amino-terminal 343 residues of these isoforms are identical in sequence, and both isoforms contain the collagen superfamily domain and RNA-binding subdomain we have identified between residues 321–339. Importantly, although we found a low abundance of isoform II transcripts in Huh-7.5 cells (Fig. 2B), only isoform I (49.7 kDa) was detected in immunoblots of Huh7.5-TLR3 and PH5CH8 cells, and not isoform II (39.6 kDa) or III (42.9 kDA).

The positively-charged carboxy-terminal region of the collagen superfamily domain is required for the association of MSR1 with acetylated LDL [41]. Its sequence is highly conserved among mammalian species with the exception, interestingly, of the chimpanzee (Fig. 5A). In addition to the 4 positively charged residues (Arg^325^, Lys^332^, Lys^335^ and Lys^338^) present in human MSR1, this subdomain contains a conserved negatively-charged residue (Glu^337^). Previous studies suggest this domain assumes a collagen-like, triple-helical conformation at pH>4.5, stabilized in part by electrostatic interactions of Glu^337^ with one of the conserved Lys residues [34]. This leaves the remaining unpaired basic residues available for intermolecular interactions with ligands, including association with the negatively-charged sugar-phosphate backbone of dsRNA. MSR1 bound to acetylated LDL is internalized through receptor-mediated endocytosis, dissociating under acidic conditions within the endosome due to the loss of ion pairing between Glu^337^ and the conserved Lys residues within the collagen-like domain [34], [42]. We presume that MSR1 functions similarly in the uptake of dsRNA from the extracellular milieu. This may explain why inhibitors of endosomal acidification block TLR3-mediated antiviral responses, as we have shown previously for Huh7.5-TLR3 cells [4], and inhibit the co-immunoprecipitation of HCV RNA with TLR3 in infected Huh7.5-TLR3 cells (Fig. 6D).

An important observation to emerge from these studies is that hepatocytes are capable of sensing HCV infection in adjacent cells, and that MSR1 mediates this response by acting as a carrier of replication intermediates (presumably dsRNA) from the extracellular milieu to endosomally expressed TLR in uninfected cells. While it is often assumed that TLR3 expressed within parenchymal cells such as hepatocytes may sense virus infection in neighboring cells, we demonstrated this formally in co-cultures of HCV-nonpermissive, TLR3-competent cells (293FT or PH5CH8 cells) and infected Huh-7.5 cells that are deficient in both TLR3 and RIG-I sensing of HCV infection [17], [26] (Figs. 6 and 7). We show that this results in a localized antiviral effect, restricting the replication of virus in the co-cultured cells, and that it is dependent upon MSR1 expression in the uninfected cells since it can be blocked by RNAi-mediated depletion of MSR1 (Fig. 7B).

These observations have important implications for the pathogenesis of chronic hepatitis C. For reasons that are unclear, only a small fraction of hepatocytes appear to be infected with HCV in these patients [25]. Two-photon immunofluorescence microscopy of frozen sections of infected human liver tissue has revealed clusters of infected cells, identified either by detection of HCV-specific antigens or dsRNA replication intermediates, typically surrounded by greater numbers of uninfected cells [25]. The presence of these discreet foci of infection suggests that the spread of virus is actively restricted within the liver. The data we present here suggest a model in which TLR3 mediates the establishment of an antiviral state in uninfected cells adjacent to those that are infected in a process that is facilitated by the dsRNA-scavenging actions of MSR1. Such a model also explains why HCV infection induces ISG expression within the liver, despite its ability to disrupt both RIG-I and TLR3 responses by NS3/4A-mediated cleavage of the RIG-I adaptor molecule, MAVS [15], [16], and the TLR3 adaptor molecule, TICAM-1 (TRIF), within infected cells [4], [18]. TLR3 sensing of HCV infection is not likely to be restricted to neighboring hepatocytes, as we have demonstrated here, but may also occur in tissue-resident macrophages (Kupffer cells) or monocyte-macrophages recruited to the site of infection. TLR7 expressed within plasmacytoid dendritic cells (pDCs) may also sense infection in other cells [43]. While less robust on a single cell level than in these “professional” innate immune cells, TLR3-mediated antiviral responses in the very large number of parenchymal hepatocytes exposed to HCV may nonetheless make a substantial contribution overall to the induction of intrahepatic ISG responses observed in patients with chronic hepatitis C [24].

## Materials and Methods

### Cells

Huh-7.5 cells [44] were a gift from Charles Rice (Rockefeller University, NY). Huh-7.5 cells engineered to express either TLR3 or the TLR3 mutants ΔTIR, H539E or N541A have been described previously [4]. 293FT cells, human embryonic kidney cells transformed with SV40 T antigen, were purchased from Invitrogen (Carlsbad, CA). 293-hTLR3 cells (engineered to over-express human TLR3) were purchased from InvivoGen. The non-neoplastic T-antigen immortalized hepatocyte cell line PH5CH8 has been described previously [30], [45]. These cells were cultured in Dulbecco's modified Eagle's medium (Invitrogen) supplemented with 10% fetal bovine serum. Blasticidin (2 µg/ml) or G418 (0.3 mg/ml) was added for the selection of cells exogenously expressing TLR3-Flag, Myc-MSR1 and related mutants. G418 (0.3 mg/ml) was added for the selection of HCV RNA replicon colonies.

### Virus

Two strains of HCV were used in these studies: the genotype 2a JFH-1 virus [46], and HJ3-5, a cell culture-adapted genotype 1a/2a chimeric virus containing the structural proteins of the genotype 1a H77 virus placed within the background of JFH-1 virus [47], [48]. HJ3-5/GLuc2A is a derivative of HJ3-5 containing the *Gaussia princeps* luciferase (GLuc) coding sequence fused to the foot-and-mouth disease virus (FMDV) 2A sequence and inserted between p7 and NS2 of HJ3-5 virus [49]. Cells were infected at an m.o.i. of 1. GLuc activity in supernatants was measured by BioLux *Gaussia* Luciferase Assay Kit (New England Biolabs, Ipswich, MA) using a Synergy2 multi-mode microplate reader (BioTek, Winooski, VT). HJ3-5/5A-YFP is another derivative of HJ3-5 containing yellow fluorescent protein (YFP) coding sequence fused to NS5A sequence [35].

### Plasmids

ptat2ANeoH77S [27] contains the *tat* protein, 15 amino acids of the FMDV 2A protein and neomycin phosphotransferase (Neo^R^) downstream of HCV internal ribosome entry site (IRES) and the full-length H77S (genotype 1a) polyprotein-coding sequence downstream of the encephalomyocarditis virus IRES. pIFN-β-Luc and pPRDII-Luc have been described previously [50], [51]. pIFN-β-mCherry, which expresses the mCherry fluorescent protein under transcriptional control of the IFN-β promoter, was constructed by replacing the firefly luciferase sequence in pIFN-β-Luc with the mCherry sequence. pJFH1-T3 was constructed by introducing a T3 promoter downstream of the HCV 3′UTR in pJFH1 [46].

pCX4neo/Myc-MSR1 and pCX4bsr/Myc-MSR1 were constructed from the retroviral vectors pCX4neo and pCX4br [52], which contain the resistance gene for neomycin and blasticidin respectively. A DNA fragment encoding MSR1 (accession no. NM_138715) was amplified from cDNA obtained from Huh-7 cell DNA by PCR using PrimeSTAR HS DNA polymerase (TaKaRa) and primers with *Sph*I (forward) and the *Not*I (reverse) recognition sites that were designed to enable expression of the MSR1 ORF. The DNA was cloned into the *Sph*I and *Not*I sites of pCX4neo/Myc and pCX4bsr/Myc, fusing MSR1 sequence to Myc. Mutations within the Myc-MSR1 sequence were subsequently constructed by PCR mutagenesis as previously described [53]. The nucleotide sequences of these vectors were confirmed by DNA sequencing. Cells stably expressing Myc-MSR1 were prepared as previously described [54].

### Synthetic HCV dsRNA

pJFH1-T3 was linearized by either *Xba*I or *Eco*RI to provide templates for synthesis of positive- or negative-stranded HCV RNA using T7 or T3 MEGAscript kits (Ambion, Austin, TX). Positive- and negative-stranded HCV RNA products were annealed to produce dsRNA by heating at 70°C for 10 minutes followed by slow cooling to room temperature. The annealed product was assayed for sensitivity to S1 nuclease (Promega, Madison, WI) to confirm that it was double-stranded.

### Poly(I:C)

High molecular weight (HMW) poly(I:C) was purchased from Invivogen (San Diego, CA). Cells were exposed to a concentration of 50 µg/ml for 6 hrs unless otherwise stated. Fluorescein-labeled HMW poly(I:C) (Invivogen) was used to monitor dsRNA uptake by cells. Cells were mock-exposed or exposed to 10 µg/ml fluorescein-labeled poly (I:C) for 8, 16 or 24 hrs, then harvested by trypsinization, washed twice in phosphate buffered saline (PBS) and fixed for 15 minutes in 4% paraformaldehyde. After additional washing in 1× PBS, the fluorescence intensity of cell populations was analyzed using a Beckman Coulter (Dako) CyAn flow cytometer.

### Promoter reporter assays

IFN-β and NF-κB-dependent promoter activities were assayed using firefly luciferase reporters, pIFN-β-Luc or pPRDII-Luc, with the reporter plasmid pRL-CMV used as an internal control for transfection efficiency as previously described [55]. A Turner Designs Luminometer Model TD-20/20 (Promega, Madison, WI) was used to measure luciferase activity. Data shown represent means ± s.d. from three independent transfection experiments.

### Immunoblot analysis

Preparation of cell lysates and SDS-PAGE were carried out as previously described [56]. Total protein was transferred to Immobilon-psq PVDF membranes (Millipore, Billerica, MA) using a Trans-blot SD semi-dry transfer cell (Bio-Rad, Hercules, CA). Primary antibodies included anti-Flag (M2; Sigma, St Louis, MO), anti-Myc (9B11; Cell Signaling, Danvers, MA), anti-ISG15 (H-150; Santa Cruz Biotechnology Inc., Santa Cruz, CA), anti-MSR1 (H-190; Santa Cruz Biotechnology Inc.), and anti-β-actin antibody (AC-15; Sigma). Secondary antibodies were IRDye-conjugated anti-mouse IgG and anti-rabbit IgG (LI-COR Biosciences, Lincoln, NE). Immunocomplexes were detected with an Odyssey infrared imaging system (LI-COR Biosciences).

### Quantitative RT-PCR analysis

Total cellular RNA was isolated using the RNeasy mini kit (Qiagen, Valencia, CA). The iScript one-step RT-PCR kit with SYBR Green and CFX96 real-time system (Bio-Rad) were used to quantify the abundance of IFN-β, ISG56, GAPDH mRNA or HCV RNA. We used the following forward and reverse primer sets: IFN-β, 5′-GTGCCTGGACCATAGTCAGAGTGG-3′ (forward), 5′-TGTCCAGTCCCAGAGGCACAGG-3′ (reverse); ISG56, AAGCTTGAGCCTCCTTGGGTTCGT-3′ (forward), 5′-TCAAAGTCAGCAGCCAGTCTCAGG-3′ (reverse); GAPDH [53], HCV, 5′-CATGGCGTTAGTATGAGTGTCGT-3′ (forward), 5′-CCCTATCAGGCAGTACCACAA-3′ (reverse). IFN-β, ISG56 and HCV RNAs were normalized to GAPDH mRNA. Results shown represent means ± s.d. from three independent experiments.

### Co-immunoprecipitation of Flag-TLR3 or Myc-MSR1 with HCV RNA

Total cell lysates were prepared using lysis buffer (PBS containing 0.2% Triton X-100, RNase inhibitor and protease inhibitor cocktail), followed by immunoprecipitation with anti-Flag or anti-Myc antibodies using protein G sepharose (GE healthcare). RNAs were extracted from the immunoprecipitates using Trizol (Invitrogen), and assayed for HCV RNA by RT-PCR using the Superscript III One-step RT-PCR system (Invitrogen) followed by agarose gel electrophoresis.

### MSR1-depleted cells and Myc-MSR1 expression

Short hairpin RNA (shRNA) targeting MSR1 (shMSR1, 5′-GCATTGATGAGAGTGCTATTG-3′) or non-targeting control shRNA (Sigma; Mission shRNA SHC-002) were introduced into Huh7.5-TLR3 or PH5CH8 cells by lentiviral transfer. MSR1-depleted Huh7.5-TLR3/shMSR1 and PH5CH8/shMSR1 and related control cells, Huh7.5-TLR3/shNT or PH5CH8/shNT cells, were selected by addition of puromycin (5 µg/ml) to the cell culture medium. MSR1 expression was reconstituted in MSR1-depleted cells by retroviral transfer of the Myc-MSR1 sequence in pCX4neo Myc-MSR1, which lacks the shMSR1 target sequence within the 5′UTR of MSR1 mRNA [57]. Cells stably expressing Myc-MSR1 were selected by growth in G418 (0.3 mg/ml).

For analysis of the RNA-binding domain in MSR1, pCX4neoMyc-MSR1 was subjected to PCR-based mutagenesis using standard methods, with the sequence of the manipulated regions of the plasmid confirmed by DNA sequencing. Cell surface expression of MSR1 and related mutants was analyzed by flow cytometry. MSR1 has a transmembrane domain between aa 51–73, with its carboxyl terminus exposed to the extracellular environment. For detection of MSR1 on the cell surface, non-permeabilized cells were fixed with 2% paraformaldehyde followed by incubation with anti-MSR1 antibody (Santacruz, H-190) for 1 h at room temperature. Cells were washed three times with PBS, and incubated with R-phycoerythrin-conjugated anti-rabbit IgG secondary antibody (Jackson ImmunoResearch) for 30 min at room temperature. Fluorescent intensity of cells was determined using a FACScan (Becton Dickinson) flow cytometer.

### Measurements of apoptosis in infected cells

Huh-7.5 cells were infected with HJ3-5/GLuc2A virus at an m.o.i. of 0.03, or mock-infected, and cultured for 4 days. As a positive control, cells were treated with 1 µM staurosporine for 6 hrs. Cells were harvested by trypsinization, washed twice in PBS and fixed in 4% paraformaldehyde, then stained for cleaved caspase 3 and HCV core protein as described previously [37]. DNA fragmentation was analyzed by terminal deoxynucleotidyltransferase-mediated dUTP-biotin nick end-labeling (TUNEL) system (Promega, Madison, WI). Positive cells were quantified by flow cytometry as described previously [37].

### Statistical methods

Statistical comparisons were carried out using Student's T test unless otherwise noted. Calculations were made with Excel 2008 for Mac (Microsoft) or Prism V for Mac OS X (GraphPad Software).

## Supporting Information

Figure S1 **Replicon colony formation assay demonstrates that TLR3 expression restricts HCV replication.** Ten µg RNA, synthesized in vitro from linearized ptat2AneoH77S DNA using a T7 MEGAscript kit (Ambion), were electroporated into Huh7.5-TLR3, -ΔTIR, -H539E or -N541A cells in a 4-mm cuvette by pulsing once at 400 V, 250 µF, and infinite Ω in a BioRad Gene Pulser Xcell apparatus. The cells were then cultured in G418 (0.3 mg/ml) for 3 weeks, and surviving cell colonies stained with Coomassie brilliant blue (0.06% in 50% methanol-10% acetic acid).
(TIF)

Figure S2 **TLR3 preferentially senses very high molecular weight poly(I:C).** (**A**) To determine whether TLR3 discriminates between dsRNA of different lengths corresponding to the size of viral genomes, we studied two dsRNA surrogates, low-molecular weight (LMW) and high-molecular weight (HMW) poly(I:C), that are between 0.2–1.0 and 1.5–8 kilobase pairs, respectively. (**B**) Both LMW and HMW poly(I:C) stimulated IFN-β promoter activity in a dose-dependent manner when added to the medium bathing (left) Huh-7.5 cells engineered to express wt TLR3 (Huh7.5-TLR3 cells), but not (right) Huh7.5-ΔTIR cells that express a defective TLR3 lacking the TIR domain and thus incapable of signaling. Importantly, however, HMW poly(I:C) was 300-fold more active than LMW poly(I:C) on a molar basis in stimulating IFN-β promoter activity. (**C**) This was reflected in significantly greater induction of ISG56 mRNA expression by HMW vs. LMW poly(I:C) in Huh7.5-TLR3 cells or PH5CH8 cells that naturally express TLR3. (**D**) At comparable concentrations, HMW poly(I:C) was also more active than LMW poly(I:C) in stimulating ISG15 protein expression in Huh7.5-TLR3 cells. Note the absence of ISG15 expression induced by either poly(I:C) in Huh7.5-H539E cells that express an inactive TLR3 mutant that is defective in dsRNA binding. (**E**) Similar differences in poly(I:C) induction of ISG15 protein expression were observed in PH5CH8 cells. Note that ISG15 expression was reduced by shRNA knockdown of TLR3 in these cells. Collectively, these results suggest that very lengthy dsRNA, such as viral replication intermediates, are more powerful inducers of TLR3-mediated antiviral responses than dsRNAs under 1 kb in length. While the mechanistic basis of this is uncertain, one possibility is that the greater signaling strength derives from progressive recruitment of multiple TLR3 ectodomains aligned along a single dsRNA molecule.
(TIF)

Figure S3 **Induction of IFN-β** **promoter activity in 293-hTLR3/IFN-β** **-mCherry cells co-cultured with HCV-infected Huh-7.5 cells.** (**A**) Human 293-hTLR3/IFN-β-mCherry cells transduced to overexpress TLR3 and the IFN-β-mCherry reporter were co-cultured with infected or uninfected Huh-7.5 cells using the same general experimental design as in the experiment shown in Fig. 6A in the main manuscript. (**B**) Immunofluorescence microscopy demonstrating induction of mCherry expression in 293-hTLR3/IFN-β-mCherry + Huh-7.5 cell co-cultures upon stimulation with poly(I:C) or infection with HJ3-5/NS5A-YFP virus. HCV replication was visualized by YFP expression and is observed in cells adjacent to those expressing mCherry in the two-color merged images at the bottom. Nuclei were visualized by DAPI counterstain.
(TIF)

Figure S4 **Absence of apoptosis in HJ3-5/GLuc2A-infected cells.** Analysis of cleaved caspase 3 and HCV core protein (top row) and DNA fragmentation by TUNEL assay (bottom row) in Huh-7.5 cells at 4 d following mock infection or infection with HJ3-5/GLuc2A virus at a m.o.i. of 0.03. Cells treated with 1 µM staurosporine for 3 hrs are shown as a positive control for apoptosis induction.
(TIF)

## References

[1] Lemon SM, Walker C, Alter MJ, Yi M (2007) Hepatitis C viruses. In: Knipe DM, Howley PM, Griffin DE, Martin MA, Lamb RA, et al.., editors. Fields Virology, 5th Ed. Philadelphia: Lippincott Williams & Wilkins. pp. 1253–1304.

[2] Lemon SM (2010) Induction and evasion of innate antiviral responses by hepatitis C virus. J Biol Chem 285: 22741–22747. 20457596 10.1074/jbc.R109.099556 PMC2906263

[3] Yoneyama M, Kikuchi M, Natsukawa T, Shinobu N, Imaizumi T, et al (2004) The RNA helicase RIG-I has an essential function in double-stranded RNA-induced innate antiviral responses. Nat Immunol 5: 730–737. 15208624 10.1038/ni1087

[4] Wang N, Liang Y, Devaraj S, Wang J, Lemon SM, et al (2009) Toll-like receptor 3 mediates establishment of an antiviral state against hepatitis C virus in hepatoma cells. J Virol 83: 9824–9834. 19625408 10.1128/JVI.01125-09 PMC2747996

[5] Kato H, Takeuchi O, Sato S, Yoneyama M, Yamamoto M, et al (2006) Differential roles of MDA5 and RIG-I helicases in the recognition of RNA viruses. Nature 441: 101–105. 16625202 10.1038/nature04734

[6] Alexopoulou L, Holt AC, Medzhitov R, Flavell RA (2001) Recognition of double-stranded RNA and activation of NF-kappaB by Toll-like receptor 3. Nature 413: 732–738. 11607032 10.1038/35099560

[7] Kawai T, Akira S (2011) Toll-like receptors and their crosstalk with other innate receptors in infection and immunity. Immunity 34: 637–650. 21616434 10.1016/j.immuni.2011.05.006

[8] Hornung V, Ellegast J, Kim S, Brzozka K, Jung A, et al (2006) 5′-Triphosphate RNA is the ligand for RIG-I. Science 314: 994–997. 17038590 10.1126/science.1132505

[9] Vercammen E, Staal J, Beyaert R (2008) Sensing of viral infection and activation of innate immunity by toll-like receptor 3. Clin Microbiol Rev 21: 13–25. 18202435 10.1128/CMR.00022-07 PMC2223843

[10] Xagorari A, Chlichlia K (2008) Toll-like receptors and viruses: induction of innate antiviral immune responses. Open Microbiol J 2: 49–59. 19088911 10.2174/1874285800802010049 PMC2593046

[11] Johnsen IB, Nguyen TT, Ringdal M, Tryggestad AM, Bakke O, et al (2006) Toll-like receptor 3 associates with c-Src tyrosine kinase on endosomes to initiate antiviral signaling. EMBO J 25: 3335–3346. 16858407 10.1038/sj.emboj.7601222 PMC1523188

[12] de Bouteiller O, Merck E, Hasan UA, Hubac S, Benguigui B, et al (2005) Recognition of double-stranded RNA by human toll-like receptor 3 and downstream receptor signaling requires multimerization and an acidic pH. J Biol Chem 280: 38133–38145. 16144834 10.1074/jbc.M507163200

[13] Saito T, Owen DM, Jiang F, Marcotrigiano J, Gale M Jr (2008) Innate immunity induced by composition-dependent RIG-I recognition of hepatitis C virus RNA. Nature 454: 523–527. 18548002 10.1038/nature07106 PMC2856441

[14] Loo YM, Owen DM, Li K, Erickson AL, Johnson CL, et al (2006) Viral and therpeutic control of interferon beta promoter stimulator 1 during hepatitis C virus infection. ProcNatlAcadSci USA 103: 6001–6006. 10.1073/pnas.0601523103 PMC1458687 16585524

[15] Meylan E, Curran J, Hofmann K, Moradpour D, Binder M, et al (2005) Cardif is an adaptor protein in the RIG-I antiviral pathway and is targeted by hepatitis C virus. Nature 437: 1167–1172. 16177806 10.1038/nature04193

[16] Li XD, Sun L, Seth RB, Pineda G, Chen ZJ (2005) Hepatitis C virus protease NS3/4A cleaves mitochondrial antiviral signaling protein off the mitochondria to evade innate immunity. ProcNatlAcadSci USA 102: 17717–17722. 10.1073/pnas.0508531102 PMC1308909 16301520

[17] Li K, Chen Z, Kato N, Gale M Jr, Lemon SM (2005) Distinct poly-I:C and virus-activated interferon signaling pathways in hepatocytes. J BiolChem 280: 16739–16747. 10.1074/jbc.M414139200 15737993

[18] Li K, Foy E, Ferreon JC, Nakamura M, Ferreon ACM, et al (2005) Immune evasion by hepatitis C virus NS3/4A protease-mediated cleavage of the TLR3 adaptor protein TRIF. Proc Natl Acad Sci U S A 102: 2992–2997. 15710891 10.1073/pnas.0408824102 PMC548795

[19] Targett-Adams P, Boulant S, McLauchlan J (2008) Visualization of double-stranded RNA in cells supporting hepatitis C virus RNA replication. J Virol 82: 2182–2195. 18094154 10.1128/JVI.01565-07 PMC2258944

[20] Limmon GV, Arredouani M, McCann KL, Corn Minor RA, Kobzik L, et al (2008) Scavenger receptor class-A is a novel cell surface receptor for double-stranded RNA. FASEB J 22: 159–167. 17709607 10.1096/fj.07-8348com

[21] Dieudonne A, Torres D, Blanchard S, Taront S, Jeannin P, et al (2012) Scavenger receptors in human airway epithelial cells: role in response to double-stranded RNA. PLoS One 7: e41952. 22879901 10.1371/journal.pone.0041952 PMC3413698

[22] DeWitte-Orr SJ, Collins SE, Bauer CM, Bowdish DM, Mossman KL An accessory to the ‘Trinity’: SR-As are essential pathogen sensors of extracellular dsRNA, mediating entry and leading to subsequent type I IFN responses. PLoS Pathog 6: e1000829. 10.1371/journal.ppat.1000829 PMC2847946 20360967

[23] Yamashita M, Chattopadhyay S, Fensterl V, Saikia P, Wetzel JL, et al (2012) Epidermal growth factor receptor is essential for toll-like receptor 3 signaling. Sci Signal 5: ra50. 22810896 10.1126/scisignal.2002581 PMC3431157

[24] Sarasin-Filipowicz M, Oakeley EJ, Duong FH, Christen V, Terracciano L, et al (2008) Interferon signaling and treatment outcome in chronic hepatitis C. Proc Natl Acad Sci U S A 105: 7034–7039. 18467494 10.1073/pnas.0707882105 PMC2383932

[25] Liang Y, Shilagard T, Xiao SY, Snyder N, Lau D, et al (2009) Visualizing hepatitis C virus infections in human liver by two-photon microscopy. Gastroenterology 137: 1448–1458. 19632233 10.1053/j.gastro.2009.07.050

[26] Sumpter R Jr, Loo YM, Foy E, Li K, Yoneyama M, et al (2005) Regulating intracellular antiviral defense and permissiveness to hepatitis C virus RNA replication through a cellular RNA helicase, RIG-I. J Virol 79: 2689–2699. 15708988 10.1128/JVI.79.5.2689-2699.2005 PMC548482

[27] Yi M, Lemon SM (2004) Adaptive mutations producing efficient replication of genotype 1a hepatitis C virus RNA in normal Huh7 cells. J Virol 78: 7904–7915. 15254163 10.1128/JVI.78.15.7904-7915.2004 PMC446091

[28] Li K, Li NL, Wei D, Pfeffer SR, Fan M, et al (2012) Activation of chemokine and inflammatory cytokine response in hepatitis C virus-infected hepatocytes depends on Toll-like receptor 3 sensing of hepatitis C virus double-stranded RNA intermediates. Hepatology 55: 666–675. 22030901 10.1002/hep.24763 PMC3272326

[29] Tuplin A, Evans DJ, Simmonds P (2004) Detailed mapping of RNA secondary structures in core and NS5B-encoding region sequences of hepatitis C virus by RNase cleavage and novel bioinformatic prediction methods. J GenVirol 85: 3037–3047. 10.1099/vir.0.80141-0 15448367

[30] Kato N, Ikeda M, Mizutani T, Sugiyama K, Noguchi M, et al (1996) Replication of hepatitis C virus in cultured non-neoplastic human hepatocytes. Japanese Journal of Cancer Research (Amsterdam) 87: 787–792. 10.1111/j.1349-7006.1996.tb02101.x PMC5921176 8797883

[31] Leonard JN, Ghirlando R, Askins J, Bell JK, Margulies DH, et al (2008) The TLR3 signaling complex forms by cooperative receptor dimerization. Proc Natl Acad Sci U S A 105: 258–263. 18172197 10.1073/pnas.0710779105 PMC2224197

[32] Kodama T, Freeman M, Rohrer L, Zabrecky J, Matsudaira P, et al (1990) Type I macrophage scavenger receptor contains alpha-helical and collagen-like coiled coils. Nature 343: 531–535. 2300204 10.1038/343531a0

[33] Shimakami T, Yamane D, Jangra RK, Kempf BJ, Spaniel C, et al (2012) Stabilization of hepatitis C RNA by an Ago2-miR-122 complex. Proc Natl Acad Sci U S A 109: 941–946. 22215596 10.1073/pnas.1112263109 PMC3271899

[34] Anachi RB, Siegel DL, Baum J, Brodsky B (1995) Acid destabilization of a triple-helical peptide model of the macrophage scavenger receptor. FEBS Lett 368: 551–555. 7635219 10.1016/0014-5793(95)00738-u

[35] Ma Y, Anantpadma M, Timpe JM, Shanmugam S, Singh SM, et al (2011) Hepatitis C virus NS2 protein serves as a scaffold for virus assembly by interacting with both structural and nonstructural proteins. J Virol 85: 86–97. 20962101 10.1128/JVI.01070-10 PMC3014171

[36] Li Y, Masaki T, Yamane D, McGivern DR, Lemon SM (2013) Competing and noncompeting activities of miR-122 and the 5′ exonuclease Xrn1 in regulation of hepatitis C virus replication. Proc Natl Acad Sci U S A 110: 1881–1886. 23248316 10.1073/pnas.1213515110 PMC3562843

[37] Kannan RP, Hensley LL, Evers L, Lemon SM, McGivern DR (2011) Hepatitis C virus infection causes cell cycle arrest at the level of entry to mitosis. J Virol 85: 7989–8001. 21680513 10.1128/JVI.00280-11 PMC3147967

[38] Suzuki H, Kurihara Y, Takeya M, Kamada N, Kataoka M, et al (1997) A role for macrophage scavenger receptors in atherosclerosis and susceptibility to infection. Nature 386: 292–296. 9069289 10.1038/386292a0

[39] Yew KH, Carsten B, Harrison C (2010) Scavenger receptor A1 is required for sensing HCMV by endosomal TLR-3/-9 in monocytic THP-1 cells. Mol Immunol 47: 883–893. 19914718 10.1016/j.molimm.2009.10.009

[40] Freeman M, Ashkenas J, Rees DJ, Kingsley DM, Copeland NG, et al (1990) An ancient, highly conserved family of cysteine-rich protein domains revealed by cloning type I and type II murine macrophage scavenger receptors. Proc Natl Acad Sci U S A 87: 8810–8814. 1978939 10.1073/pnas.87.22.8810 PMC55049

[41] Doi T, Higashino K, Kurihara Y, Wada Y, Miyazaki T, et al (1993) Charged collagen structure mediates the recognition of negatively charged macromolecules by macrophage scavenger receptors. J Biol Chem 268: 2126–2133. 8380589

[42] Doi T, Kurasawa M, Higashino K, Imanishi T, Mori T, et al (1994) The histidine interruption of an alpha-helical coiled coil allosterically mediates a pH-dependent ligand dissociation from macrophage scavenger receptors. J Biol Chem 269: 25598–25604. 7929263

[43] Takahashi K, Asabe S, Wieland S, Garaigorta U, Gastaminza P, et al (2010) Plasmacytoid dendritic cells sense hepatitis C virus-infected cells, produce interferon, and inhibit infection. Proc Natl Acad Sci U S A 107: 7625–7626. 20231459 10.1073/pnas.1002301107 PMC2867703

[44] Blight KJ, McKeating JA, Rice CM (2002) Highly permissive cell lines for subgenomic and genomic hepatitis C virus RNA replication. J Virol 76: 13001–13014. 12438626 10.1128/JVI.76.24.13001-13014.2002 PMC136668

[45] Ikeda M, Sugiyama K, Mizutani T, Tanaka T, Tanaka K, et al (1998) Human hepatocyte clonal cell lines that support persistent replication of hepatitis C virus. Virus Res 56: 157–167. 9783464 10.1016/s0168-1702(98)00063-x

[46] Wakita T, Pietschmann T, Kato T, Date T, Miyamoto M, et al (2005) Production of infectious hepatitis C virus in tissue culture from a cloned viral genome. Nature Medicine 11: 791–796. 10.1038/nm1268 PMC2918402 15951748

[47] Yi M, Ma Y, Yates J, Lemon SM (2007) Compensatory mutations in E1, p7, NS2, and NS3 enhance yields of cell culture-infectious intergenotypic chimeric hepatitis C virus. J Virol 81: 629–638. 17079282 10.1128/JVI.01890-06 PMC1797426

[48] Ma Y, Yates J, Liang Y, Lemon SM, Yi M (2008) NS3 helicase domains involved in infectious intracellular hepatitis C virus particle assembly. J Virol 82: 7624–7639. 18508894 10.1128/JVI.00724-08 PMC2493332

[49] Shimakami T, Yamane D, Welsch C, Hensley L, Jangra RK, et al (2012) Base-pairing between Hepatitis C Virus RNA and miR-122 3′ of its Seed Sequence is Essential for Genome Stabilization and Production of Infectious Virus. J Virol 86 (13) 7372–7383. 22532678 10.1128/JVI.00513-12 PMC3416334

[50] Lin R, Genin P, Mamane Y, Hiscott J (2000) Selective DNA binding and association with the CREB binding protein coactivator contribute to differential activation of alpha/beta interferon genes by interferon regulatory factors 3 and 7. Mol Cell Biol 20: 6342–6353. 10938111 10.1128/mcb.20.17.6342-6353.2000 PMC86109

[51] Fredericksen B, Akkaraju GR, Foy E, Wang C, Pflugheber J, et al (2002) Activation of the interferon-beta promoter during hepatitis C virus RNA replication. Viral Immunol 15: 29–40. 11952144 10.1089/088282402317340215

[52] Akagi T, Sasai K, Hanafusa H (2003) Refractory nature of normal human diploid fibroblasts with respect to oncogene-mediated transformation. Proc Natl Acad Sci U S A 100: 13567–13572. 14597713 10.1073/pnas.1834876100 PMC263854

[53] Dansako H, Naganuma A, Nakamura T, Ikeda F, Nozaki A, et al (2003) Differential activation of interferon-inducible genes by hepatitis C virus core protein mediated by the interferon stimulated response element. Virus Res 97: 17–30. 14550584 10.1016/s0168-1702(03)00218-1

[54] Naganuma A, Dansako H, Nakamura T, Nozaki A, Kato N (2004) Promotion of microsatellite instability by hepatitis C virus core protein in human non-neoplastic hepatocyte cells. Cancer Res 64: 1307–1314. 14973066 10.1158/0008-5472.can-03-2992

[55] Dansako H, Ikeda M, Ariumi Y, Wakita T, Kato N (2009) Double-stranded RNA-induced interferon-beta and inflammatory cytokine production modulated by hepatitis C virus serine proteases derived from patients with hepatic diseases. Arch Virol 154: 801–810. 19353241 10.1007/s00705-009-0375-z

[56] Dansako H, Naka K, Ikeda M, Kato N (2005) Hepatitis C virus proteins exhibit conflicting effects on the interferon system in human hepatocyte cells. Biochem Biophys Res Commun 336: 458–468. 16139243 10.1016/j.bbrc.2005.08.112

[57] Dansako H, Ikeda M, Kato N (2007) Limited suppression of the interferon-beta production by hepatitis C virus serine protease in cultured human hepatocytes. Febs J 274: 4161–4176. 17651439 10.1111/j.1742-4658.2007.05942.x

[58] Marchler-Bauer A, Lu S, Anderson JB, Chitsaz F, Derbyshire MK, et al (2011) CDD: a Conserved Domain Database for the functional annotation of proteins. Nucleic Acids Res 39: D225–229. 21109532 10.1093/nar/gkq1189 PMC3013737

