# Supplementary figures and images for: Class A Scavenger Receptor 1 (MSR1) Restricts Hepatitis C Virus Replication by Mediating Toll-like Receptor 3 Recognition of Viral RNAs Produced in Neighboring Cells
Source: PLoS Pathog. 2013 May 23;9(5):e1003345. doi: 10.1371/journal.ppat.1003345 (PMC3662657; doi:10.1371/journal.ppat.1003345)

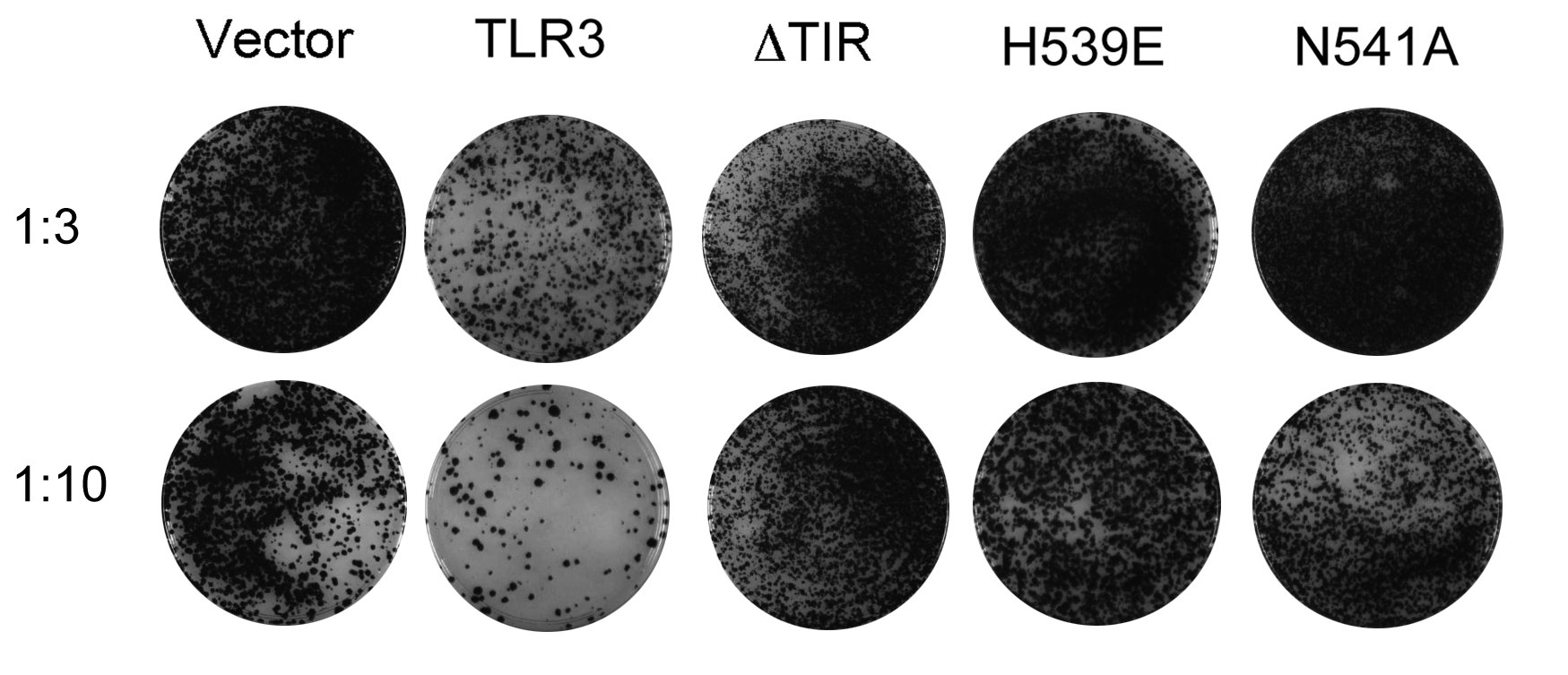

Supplement: Figure S1 — Replicon colony formation assay demonstrates that TLR3 expression restricts HCV replication. Ten µg RNA, synthesized in vitro from linearized ptat2AneoH77S DNA using a T7 MEGAscript kit (Ambion), were electroporated into Huh7.5-TLR3, -ΔTIR, -H539E or -N541A cells in a 4-mm cuvette by pulsing once at 400 V, 250 µF, and infinite Ω in a BioRad Gene Pulser Xcell apparatus. The cells were then cultured in G418 (0.3 mg/ml) for 3 weeks, and surviving cell colonies stained with Coomassie brilliant blue (0.06% in 50% methanol-10% acetic acid). (TIF) [file ppat.1003345.s001.tif]

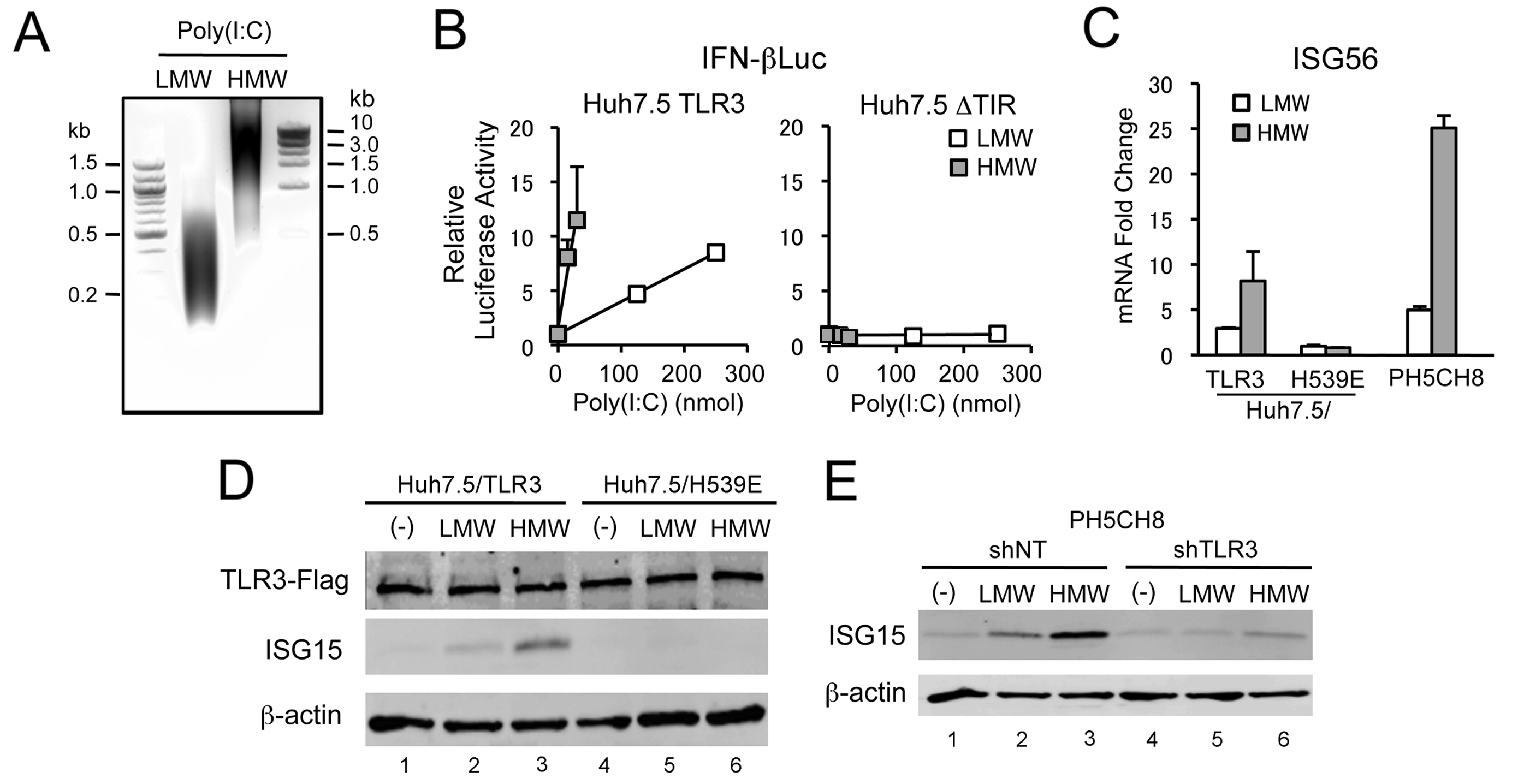

Supplement: Figure S2 — TLR3 preferentially senses very high molecular weight poly(I:C). (A) To determine whether TLR3 discriminates between dsRNA of different lengths corresponding to the size of viral genomes, we studied two dsRNA surrogates, low-molecular weight (LMW) and high-molecular weight (HMW) poly(I:C), that are between 0.2–1.0 and 1.5–8 kilobase pairs, respectively. (B) Both LMW and HMW poly(I:C) stimulated IFN-β promoter activity in a dose-dependent manner when added to the medium bathing (left) Huh-7.5 cells engineered to express wt TLR3 (Huh7.5-TLR3 cells), but not (right) Huh7.5-ΔTIR cells that express a defective TLR3 lacking the TIR domain and thus incapable of signaling. Importantly, however, HMW poly(I:C) was 300-fold more active than LMW poly(I:C) on a molar basis in stimulating IFN-β promoter activity. (C) This was reflected in significantly greater induction of ISG56 mRNA expression by HMW vs. LMW poly(I:C) in Huh7.5-TLR3 cells or PH5CH8 cells that naturally express TLR3. (D) At comparable concentrations, HMW poly(I:C) was also more active than LMW poly(I:C) in stimulating ISG15 protein expression in Huh7.5-TLR3 cells. Note the absence of ISG15 expression induced by either poly(I:C) in Huh7.5-H539E cells that express an inactive TLR3 mutant that is defective in dsRNA binding. (E) Similar differences in poly(I:C) induction of ISG15 protein expression were observed in PH5CH8 cells. Note that ISG15 expression was reduced by shRNA knockdown of TLR3 in these cells. Collectively, these results suggest that very lengthy dsRNA, such as viral replication intermediates, are more powerful inducers of TLR3-mediated antiviral responses than dsRNAs under 1 kb in length. While the mechanistic basis of this is uncertain, one possibility is that the greater signaling strength derives from progressive recruitment of multiple TLR3 ectodomains aligned along a single dsRNA molecule. (TIF) [file ppat.1003345.s002.tif]

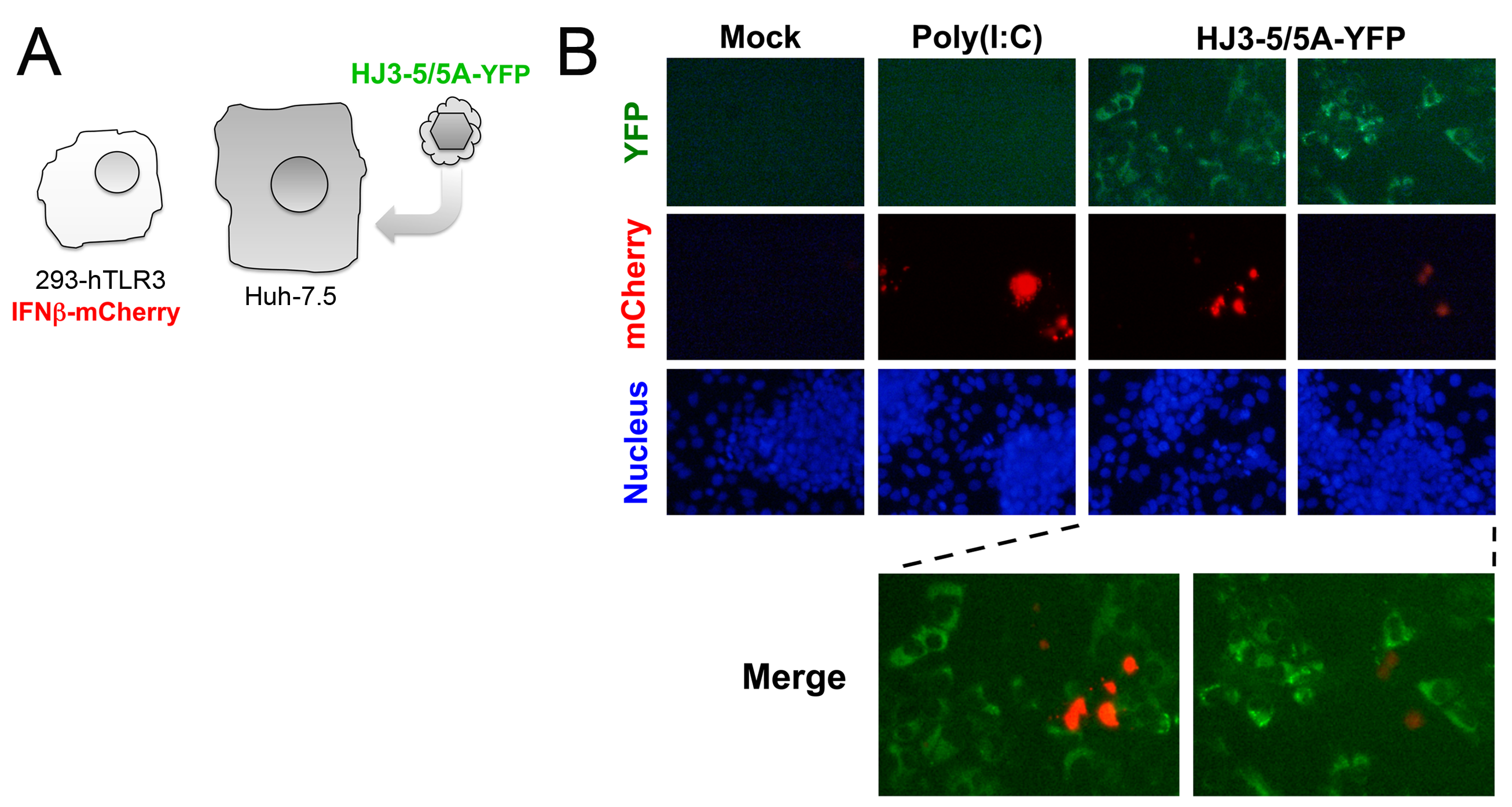

Supplement: Figure S3 — Induction of IFN-β promoter activity in 293-hTLR3/IFN-β -mCherry cells co-cultured with HCV-infected Huh-7.5 cells. (A) Human 293-hTLR3/IFN-β-mCherry cells transduced to overexpress TLR3 and the IFN-β-mCherry reporter were co-cultured with infected or uninfected Huh-7.5 cells using the same general experimental design as in the experiment shown in Fig. 6A in the main manuscript. (B) Immunofluorescence microscopy demonstrating induction of mCherry expression in 293-hTLR3/IFN-β-mCherry + Huh-7.5 cell co-cultures upon stimulation with poly(I:C) or infection with HJ3-5/NS5A-YFP virus. HCV replication was visualized by YFP expression and is observed in cells adjacent to those expressing mCherry in the two-color merged images at the bottom. Nuclei were visualized by DAPI counterstain. (TIF) [file ppat.1003345.s003.tif]

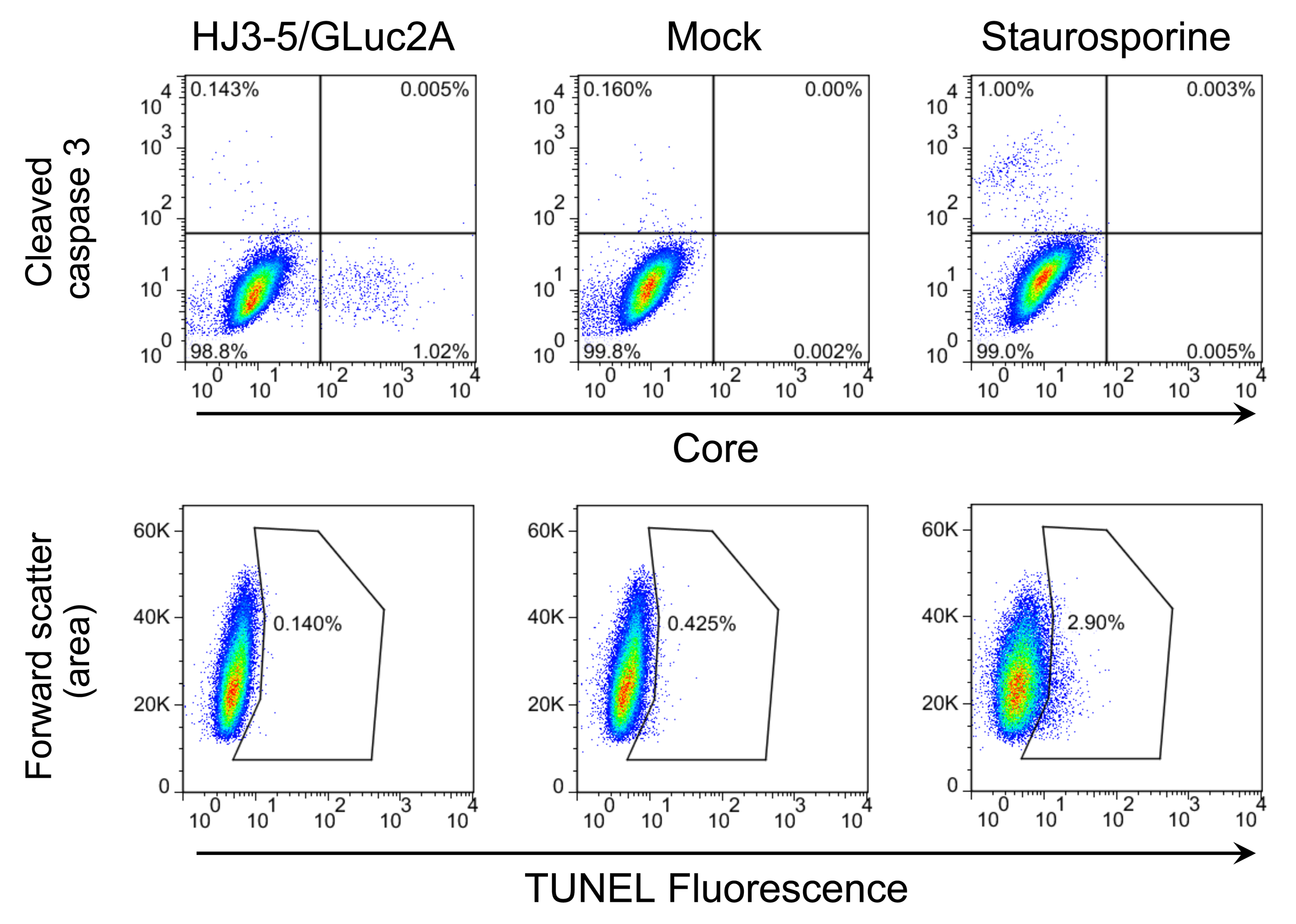

Supplement: Figure S4 — Absence of apoptosis in HJ3-5/GLuc2A-infected cells. Analysis of cleaved caspase 3 and HCV core protein (top row) and DNA fragmentation by TUNEL assay (bottom row) in Huh-7.5 cells at 4 d following mock infection or infection with HJ3-5/GLuc2A virus at a m.o.i. of 0.03. Cells treated with 1 µM staurosporine for 3 hrs are shown as a positive control for apoptosis induction. (TIF) [file ppat.1003345.s004.tif]
